# Mandibular dysmorphology due to abnormal embryonic osteogenesis in FGFR2-related craniosynostosis mice

**DOI:** 10.1242/dmm.038513

**Published:** 2019-05-30

**Authors:** Susan M. Motch Perrine, Meng Wu, Nicholas B. Stephens, Divya Kriti, Harm van Bakel, Ethylin Wang Jabs, Joan T. Richtsmeier

**Affiliations:** 1 Department of Anthropology, Pennsylvania State University, University Park, PA 16802, USA; 2 Department of Genetics and Genomic Sciences, Icahn School of Medicine at Mount Sinai, New York, NY 10029, USA

**Keywords:** Apert syndrome, Crouzon syndrome, Pfeiffer syndrome, Osteoclast, Cartilage, Transcriptome, FGFR2

## Abstract

One diagnostic feature of craniosynostosis syndromes is mandibular dysgenesis. Using three mouse models of Apert, Crouzon and Pfeiffer craniosynostosis syndromes, we investigated how embryonic development of the mandible is affected by fibroblast growth factor receptor 2 (*Fgfr2*) mutations. Quantitative analysis of skeletal form at birth revealed differences in mandibular morphology between mice carrying *Fgfr2* mutations and their littermates that do not carry the mutations. Murine embryos with the mutations associated with Apert syndrome in humans (*Fgfr2^+/S252W^* and *Fgfr2^+/P253R^*) showed an increase in the size of the osteogenic anlagen and Meckel's cartilage (MC). Changes in the microarchitecture and mineralization of the developing mandible were visualized using histological staining. The mechanism for mandibular dysgenesis in the Apert *Fgfr2^+/S252W^* mouse resulting in the most severe phenotypic effects was further analyzed in detail and found to occur to a lesser degree in the other craniosynostosis mouse models. Laser capture microdissection and RNA-seq analysis revealed transcriptomic changes in mandibular bone at embryonic day 16.5 (E16.5), highlighting increased expression of genes related to osteoclast differentiation and dysregulated genes active in bone mineralization. Increased osteoclastic activity was corroborated by TRAP assay and *in situ* hybridization of *Csf1r* and *Itgb3*. Upregulated expression of *Enpp1* and *Ank* was validated in the mandible of *Fgfr2^+/S252W^* embryos, and found to result in elevated inorganic pyrophosphate concentration. Increased proliferation of osteoblasts in the mandible and chondrocytes forming MC was identified in *Fgfr2^+/S252W^* embryos at E12.5. These findings provide evidence that FGFR2 gain-of-function mutations differentially affect cartilage formation and intramembranous ossification of dermal bone, contributing to mandibular dysmorphogenesis in craniosynostosis syndromes.

This article has an associated First Person interview with the joint first authors of the paper.

## Abstract (precis)

**Summary:** FGFR2 gain-of-function mutations differentially affect cartilage formation and intramembranous ossification of dermal bone, resulting in abnormal embryonic osteogenesis of the mandible.

## INTRODUCTION

The mandible has been used as a model for investigating how complex morphological structures arise during development and how they are altered during evolution (Atchley and Hall, 1991), providing insight into how the spatial and temporal organization underlying the development of a separate morphological component assimilates into a functioning whole. Each hemimandible (or dentary) is composed of two functional areas that are mineralized proximate to Meckel's cartilage (MC): the anterior body (tooth-bearing portion of the hemimandible) and ramus [containing three prominent processes: coronoid process on the dorsal aspect, the condylar (or condyloid) process caudally, and the angular process caudoventrally]. Mandible and maxilla are dermal bones derived from neural crest cells that migrate to the first pharyngeal arch and are the result of complex developmental patterning (Couly et al., 1996; Depew et al., 2002; Frisdal and Trainor, 2014; Noden, 1983). Together they form the lower and upper jaws of the facial skeleton, the synchronous development and proper occlusion of which is necessary for feeding, respiration and craniofacial morphogenesis.

Mutations within fibroblast growth factor receptor 2 (FGFR2) are responsible for aberrant signaling within the FGF-signaling pathway resulting in midface developmental anomalies that are features of Apert, Crouzon, Pfeiffer, Beare-Stevenson cutis gyrata, Jackson-Weiss and Bent Bone Dysplasia syndromes (Azoury et al., 2017; Cohen and MacLean, 2000; Cunningham et al., 2007). These complex conditions involve the premature fusion of one or more cranial sutures and midfacial dysgenesis and are often associated with other skeletal and soft tissue abnormalities (Flaherty et al., 2016; Heuzé et al., 2014). Midfacial dysgenesis can be severe but is variable within and across FGFR2-related craniosynostosis syndromes. Surgical correction and reconstruction are adaptable, targeting the midfacial skeleton, dental arcade, choanae and/or airway, often requiring significant and multiple reconstructive procedures. The mandible, the major skeletal element of the lower face, is an important consideration in surgical planning and orthodontic management in craniosynostosis syndromes to address severe anomalies that affect mastication and airway anomalies.

Apert, Crouzon and Pfeiffer syndromes (MIM #101200, MIM #123500, MIM #101600, respectively) are autosomal dominant conditions sharing many phenotypic similarities, including premature suture closure, abnormal facies, exophthalmos, midfacial retrusion, dental malocclusion of varying intensities, cranial base anomalies and dysmorphic mandibles, the configuration of which is discordant with the upper jaw (Cohen and MacLean, 2000). Although the mandible is not well-studied in these syndromes, adult mandibular morphology in Apert patients is usually reported as intrinsically normal, and detected differences in mandibular length are thought to be secondary to midfacial dysgenesis (Lemire, 2000; Wink et al., 2013). The apparent mandibular prognathism is thought to be relative, a condition resulting from anomalies of the cranial base and severe retrusion of the midface (Costaras-Volarich and Pruzansky, 1984). Why the degree and nature of developmental anomalies of the lower face would be different from the midface when mandible and maxillae are both dermal bones of neural crest origin derived from the first pharyngeal arch is not clear.

We have previously reported statistical differences in craniofacial bone morphology, brain morphology, soft tissue and negative space (nasopharynx, inner ear) volumes, and morphological integration of brain and skull in mouse models for Apert and Crouzon/Pfeiffer syndromes relative to their respective littermates that do not carry the mutation and show no phenotypic effects (Aldridge et al., 2010; Holmes et al., 2018; Martínez-Abadías et al., 2013; Motch Perrine et al., 2014, 2017). However, investigations of the mandible have not been included in any of these studies. To test the hypothesis that FGFR2 mutations causative for craniosynostosis syndromes target processes and mechanisms of mandibular genesis, we present data on the developmental and morphological consequences of three unique FGFR2 mutations associated with syndromic craniosynostosis in the mandible of the mouse. We performed quantitative morphometric analysis of 3D micro computed tomography (µCT) image data of three mouse models with differing activating *Fgfr2* mutations to determine the differential effects of these mutations on the mandible: two Apert syndrome mouse models, *Fgfr2^+/S252W^* (Wang et al., 2005) and *Fgfr2^+/P253R^* (Wang et al., 2010) on a C57BL6/J background, and a mouse model with a mutation associated with Crouzon and Pfeiffer syndromes, *Fgfr2c^C342Y/+^* (Eswarakumar et al., 2004) on a CD1 background. These analyses revealed significant differences in mandibular morphology at postpartum day (P) 0 in all three of the mouse models. Based on these findings, we performed histological analysis on the mandible at embryonic stages. Of these three mouse models, mandibles of *Fgfr2^+/S252W^* mice showed the greatest magnitude of morphological change at P0 and histologic differences at embryonic day (E) 16.5, and were further analyzed by transcriptome analysis to reveal cellular and molecular dysregulation contributing to mandibular dysgenesis. Increased osteoclastogenesis causes abnormal bone resorption and overexpression of *Enpp1* and *Ank* that are key regulators for inorganic pyrophosphate levels inhibiting bone mineralization in *Fgfr2^+/S252W^* mandible. FGFR2 S252W mutation was associated with increased proliferation of osteoblasts and chondrocytes in the mandible as early as E12.5. We provide new information about the molecular processes affecting the mandible in FGFR2-related craniosynostosis syndromes to improve our understanding of craniofacial dysgenesis and move us closer to therapeutic approaches for patients.

## RESULTS

### Mandibular dysmorphology of FGFR2-related craniosynostosis mouse models

**Fig. 1.**
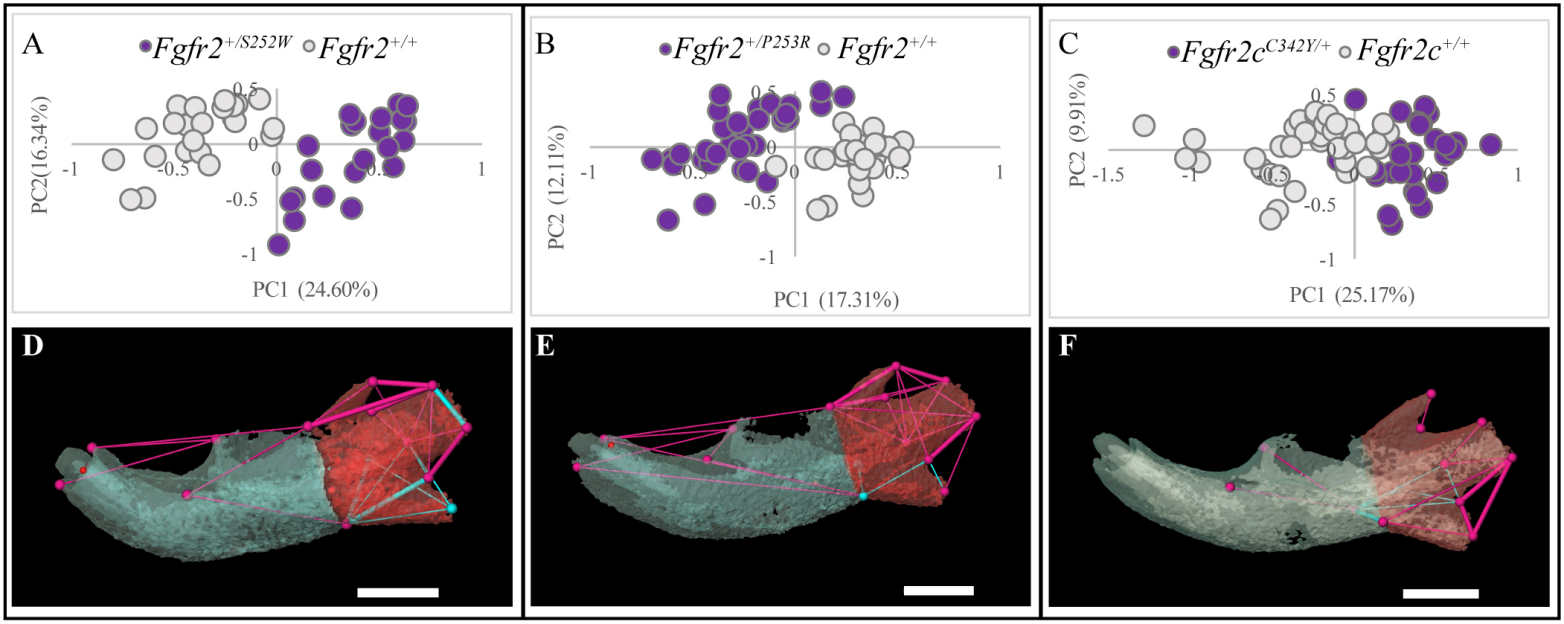
**Morphological differences in newborn (P0) mice carrying mutations associated with three FGFR2-related craniosynostosis syndromes and their unaffected littermates.** (A-F) Results of PCA of mandibles based on unique linear distances among 3D landmarks (A-C) and EDMA of landmark coordinates (D-F). Scatter plots of individual scores on first and second PC axes (PC1 and PC2) of linear distance-based PCAs of the hemimandibles of mutant and unaffected littermates of *Fgfr2^+/S252W^* and *Fgfr2^+/P253R^* Apert syndrome mouse models (A,B, respectively) and *Fgfr2c^C342Y/+^* Crouzon/Pfeiffer syndrome mouse model (C). Results of EDMA of each craniosynostosis mouse model and unaffected littermates showing linear distances within each model that are significantly different by at least 5% between mutant and unaffected littermates (D-F). Blue lines are significantly larger in mutant mice relative to unaffected littermates; fuchsia lines are significantly smaller in mutant mice. Thin lines indicate linear distances that are increased/decreased by 5-10% in mice carrying one of the *Fgfr2* mutations whereas thick lines indicate linear distances that differ by >10% between mutant and unaffected mice. The buccal aspects of the left hemimandibles of the models were used for illustration. Hemimandibles were segmented into an anterior portion (anterior body, blue) and posterior portion (ramus, red) to indicate functional areas. Scale bars: 1 mm.

The left and right hemimandibles of 182 newborn (P0) mice of each of the three craniosynostosis models of interest were analyzed morphometrically using the 3D coordinates of 32 landmarks (lms) (Fig. S1 and Table S1). Landmark datasets characterizing whole mandibles (consisting of right and left sides, 32 lms), left hemimandibles (16 lms), and right hemimandibles (16 lms) were analyzed using the same morphometric methods. Within each model, morphometric analyses compared mice carrying a specific mutation to littermates that did not carry the mutation. Results revealed a lack of asymmetry in mandibular dysmorphology in all models, such that the right and left hemimandibles were similarly affected (Table S2). For clarity of presentation, analysis of the left hemimandible is presented graphically (Fig. 1A-C).

Two morphometric methods were used. Euclidean distance matrix analysis (EDMA) (Lele and Richtsmeier, 2001) revealed significant differences in hemimandible shape between all mice carrying *Fgfr2* mutations and their respective littermates that did not carry the mutation (referred to as ‘unaffected littermates’) for the full 32 lms set (*P*≤0.001) and for all three regional landmark subsets representing major functional regions of the left dentary: left hemimandible (16 lms; *P*<0.001); ramus (10 lms; *P*<0.001); body (8 lms; *P*<0.05). Bootstrapped confidence intervals for differences in each linear distance obtained from EDMA reveal statistically significant differences in the localized patterns of mutational effects on the 3D morphology of the hemimandible (Fig. 1D-F). *Fgfr2c^C342Y/+^* mice show effects that are generally of a lesser magnitude and of a different pattern when compared to the effects of the other two mutations (Fig. 1F). There are obvious similarities in the way hemimandibles of *Fgfr2^+/S252W^* and *Fgfr2^+/P253R^* mutant mice differ from their respective unaffected littermates. However, the significant phenotypic effects of the FGFR2 S252W mutation on mandibular morphology are more numerous, of greater magnitude, and located primarily in the posterior components of the hemimandible (Fig. 1D,E).

Principal components analysis (PCA) of the scale-free shape data shows obvious separation of hemimandibles of mice carrying an *Fgfr2* mutation from their respective unaffected littermates for each of the three mutation groups (Fig. 1A-C). PCAs of shape were also conducted using linear distances estimated from the landmark coordinates that define the anterior body of the hemimandible and the ramus portion of the hemimandible (Table S1, Fig. S1). Although the anterior body of the *Fgfr2c^C342Y/+^* Crouzon/Pfeiffer syndrome mouse mandible showed a distinct morphology relative to their unaffected littermates, there was less difference in the anterior body of *Fgfr2^+/S252W^* and *Fgfr2^+/P253R^* Apert syndrome mice relative to their respective unaffected littermates (Fig. S2). All three mutation groups showed differences between mutant mice and their respective unaffected littermates in the ramus portion of the hemimandible (Fig. S2).

In every analysis, all mice carrying mutations revealed a mandibular morphology that differed from littermates that did not carry the mutation in unique ways. The finding that different *Fgfr2* gain-of-function mouse models exhibit different mandibular phenotypes is consistent with our previous work that shows that the cranial phenotype (not including the mandible) of each *Fgfr2* model is different from their respective unaffected littermates that do not carry the mutation, and that these changes vary across mouse models.

### Quantitative characterization of hemimandible bone

Bone volume and bone surface area were determined using the Material Statistics Module of Avizo 9.4 by first segmenting the left hemimandible as the region of interest from µCT scans. Bone volume and bone surface area were compared using the Mann–Whitney *U*-test and did not differ significantly between mutant and unaffected littermates in any of the three craniosynostosis mouse models (Table 1), although the *Fgfr2^+/S252W^* mice had the least bone volume of all genotypes. This is consistent with previous findings indicating no difference in bone volume of the hemimandibles in *Fgfr2^+/P253R^* mice and their controls at E15.5, E16.5, E17.5, P0 and P2 (Percival et al., 2014). Mean bone mineral density maps of the left hemimandibles reveal little variation between mutant mice and unaffected littermates across all models (Fig. S3). Cortical bone thickness was mapped in an identical manner and showed little variation (Fig. S4).

**Table 1.**
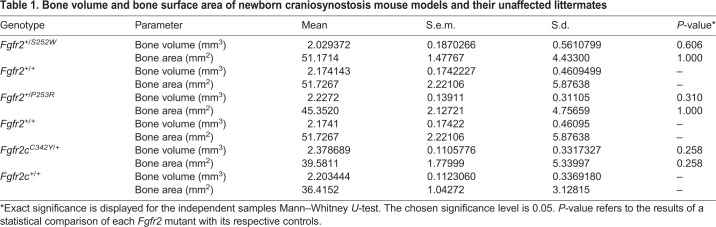
**Bone volume and bone surface area of newborn craniosynostosis mouse models and their unaffected littermates**

### Impaired microarchitecture in *Fgfr2^+/S252W^* mandible

**Fig. 2.**
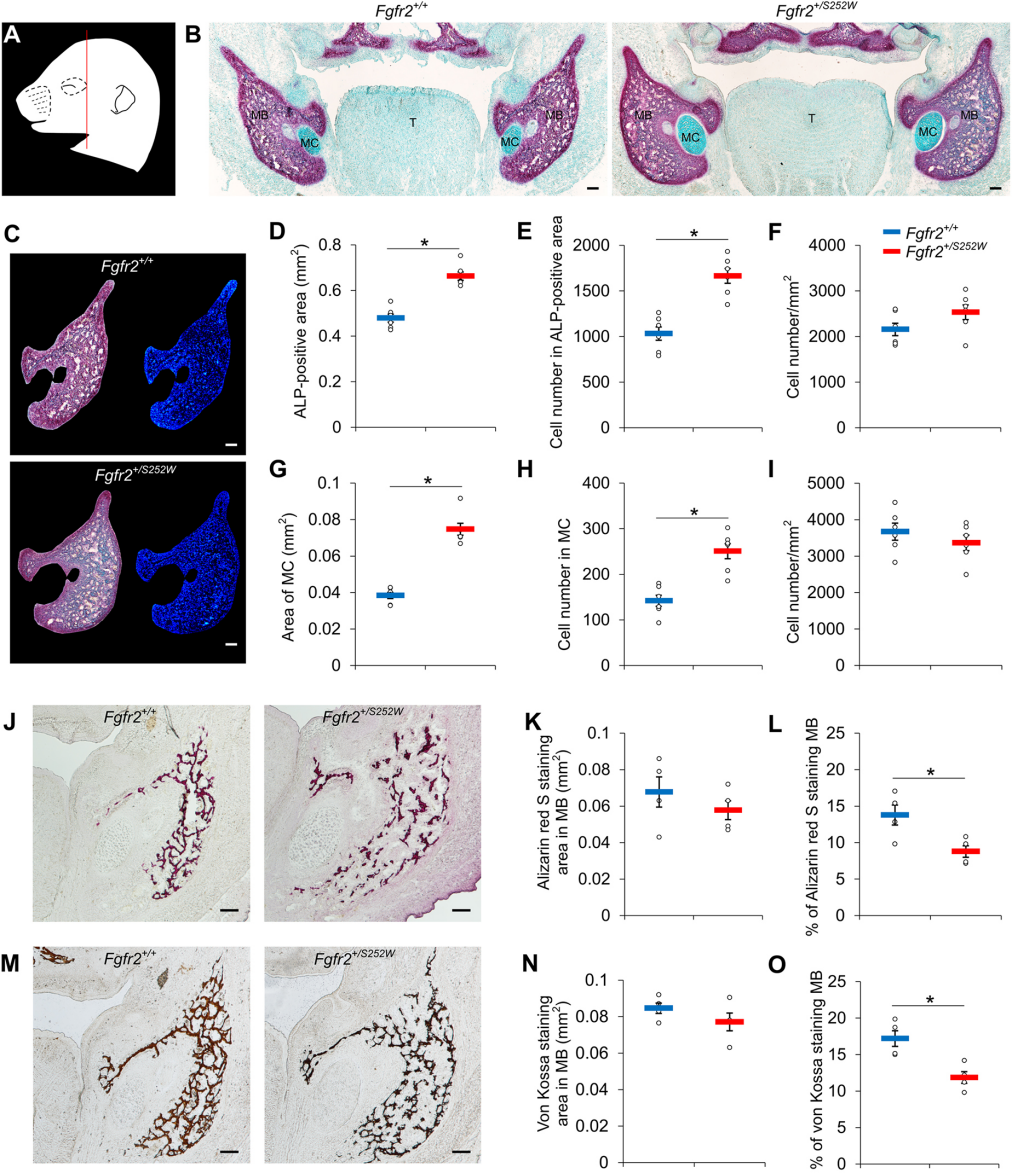
**Histological analysis of mandible of *Fgfr2^+/S252W^* embryos at E16.5.** (A) Schematic embryonic mouse head at E16.5 modified from the e-Mouse Atlas Project (http://www.emouseatlas.org/emap/eHistology). The red line indicates the location of sections used for B-O. (B) Cryosections of *Fgfr2^+/+^* and *Fgfr2^+/S252W^* embryos were stained with the ALP assay (red) and Alcian Blue. MB, mandibular bone; MC, Meckel's cartilage; T, tongue. (C) The ALP-positive regions (red) were selected to quantify the areas and numbers of nuclei stained with Hoechst 33258 (blue). (D-I) The areas (D,G), cell numbers (E,H) and cell density (F,I) in the ALP-positive regions for *Fgfr2^+/+^* (*n*=6) and *Fgfr2*^+/S252W^ (*n*=6) embryos and MC of *Fgfr2^+/+^* (*n*=6) and *Fgfr2*^+/S252W^ (*n*=6) embryos. (J,M) Alizarin Red S staining (J) and von Kossa staining (M) showing ossification in the mandible of *Fgfr2^+/+^* and *Fgfr2^+/S252W^* embryos. The areas and the percentages of the stained area in osteogenic tissue were measured for Alizarin Red S (K,L) and von Kossa (N,O) staining. Data are mean±s.e.m. *P<0.05, two-tailed Welch's *t*-test. Scale bars: 100 µm.

The mandibles of *Fgfr2^+/S252W^* Apert syndrome mice showed the greatest difference relative to their unaffected littermates in our analyses of 3D geometry and physical properties, and therefore we initially focused on this mouse model for additional analyses of histological properties of embryonic mandibular bone. We present histological analysis on the mandible at E16.5, when differences were more obvious and consistent than at earlier stages. Osteogenic tissue was defined operationally as cells that stain with alkaline phosphatase (ALP), which includes osteoprogenitors, preosteoblasts and differentiated osteoblasts (Huang et al., 2007). We performed serial coronal sections (Fig. 2A) and stained them with ALP and Alcian Blue for cartilage (Fig. 2B). *Fgfr2^+/S252W^* embryos showed significant morphological changes in the developing mandible at E16.5. Both the osteogenic tissue and MC were enlarged in *Fgfr2^+/S252W^* E16.5 embryos relative to *Fgfr2^+/+^* littermates (Fig. 2B,C), prefiguring localization of the more severe differences determined by morphometric analysis of µCT data of P0 mandibles. Quantification of ALP-positive areas and cell numbers revealed that the ALP-positive area was 38.5% larger in *Fgfr2^+/S252W^* embryos relative to *Fgfr2^+/+^* littermates (Fig. 2D) and cell number was increased by 61.3% (Fig. 2E) at E16.5, whereas the cell density was not significantly changed (Fig. 2F, *P*=0.135). The area of MC was 94.5% larger in *Fgfr2^+/S252W^* embryos relative to *Fgfr2^+/+^* littermates (Fig. 2G) and cell number was increased by 56.6% (Fig. 2H), but there was no significant change in cell density (Fig. 2I, *P*=0.354).

It has been reported that postnatal and adult *Fgfr2^S252W/+^* mutant mice showed changes in mineral apposition rate and microarchitecture of the mandible (Zhou et al., 2013). To analyze the mineralized tissues of embryonic *Fgfr2^+/S252W^* mandibles, we performed Alizarin Red S and von Kossa staining to detect calcium deposits and the presence of phosphate, respectively. In the *Fgfr2^+/+^* littermate, trabecular bone of the mandible was a well-organized network, whereas the *Fgfr2^+/S252W^* trabecular bone had a disorganized and loose structure (Fig. 2J). Quantitative analysis of Alizarin Red S staining showed that, although the total areas of mineralized tissues were not significantly changed (Fig. 2K, *P*=0.414), the amount of staining versus osteogenic area was significantly smaller in *Fgfr2^+/S252W^* embryos (Fig. 2L, *P*=0.043). Von Kossa staining revealed a similar difference in mineralization of the mandible of *Fgfr2^+/+^* and *Fgfr2^+/S252W^* embryos (Fig. 2M) and the quantification of the staining showed a similar result (Fig. 2N,O). Thus, the FGFR2 S252W mutation is associated with impaired microarchitecture affecting mandibular morphogenesis as early as E16.5.

To determine whether these changes were found in the other FGFR2-related mouse models, we performed histological analysis for mandibles of *Fgfr2^+/P253R^* and *Fgfr2c^C342Y/+^* embryos at E16.5. *Fgfr2^+/P253R^* embryos exhibited similar histological changes in the mandible of *Fgfr2^+/S252W^* embryos, including increased osteogenic tissue, MC (Fig. S5B) and disorganized mineralization pattern (Fig. S5F,J), consistent with the mandibular dysmorphology observed for these mutants at P0. *Fgfr2c^C342Y/+^* embryos showed more subtle, localized changes in mineralization in the mandible (Fig. S5H,L).

### Abnormal osteogenesis in *Fgfr2^+/S252W^* mandible

**Fig. 3.**
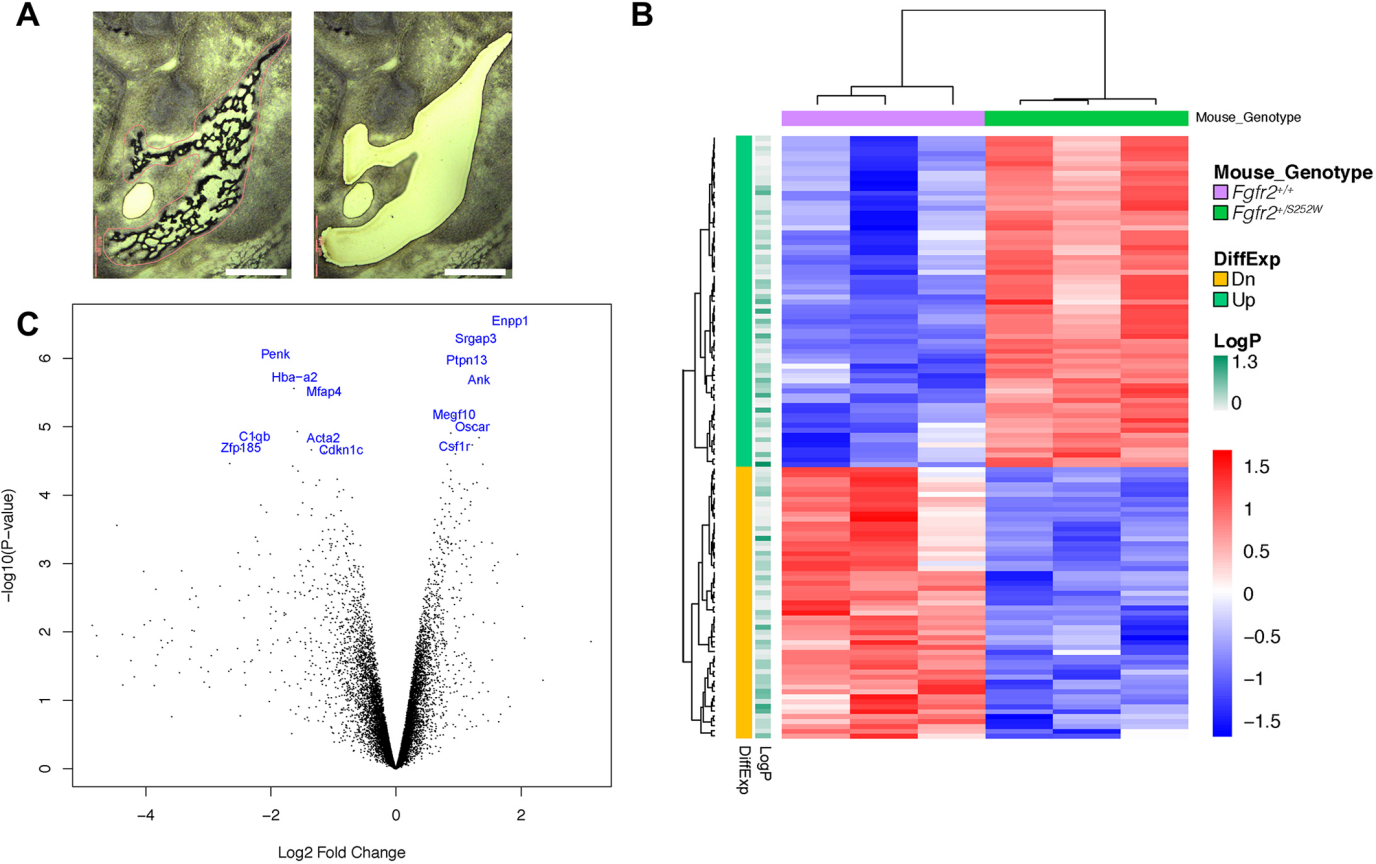
**Laser capture microdissection and RNA-seq analysis of mandibular bone of *Fgfr2^+/S252W^* embryos at E16.5.** (A) A representative mandibular region in cryosection was dissected by laser and collected for RNA-seq (left, before LCM; right, after LCM). (B) Hierarchical clustering of 122 genes significantly differentially expressed in the mandibular bone between *Fgfr2^+/S252W^* and *Fgfr2^+/+^* littermate embryos. Three biological replicates were used for each genotype. (C) Volcano plot shows *P*-values and fold changes of DEGs in the mandibular bone between *Fgfr2^+/S252W^* and *Fgfr2^+/+^* littermate embryos. Some of the most significantly differentially expressed genes [−log_10_(*P*-value)>4.5] implicated in mandibular dysmorphology are shown in blue. Scale bars: 400 µm.

To understand the molecular mechanism for the changes of bone formation in the mandible by the FGFR2 S252W mutation, we collected tissues from the mandibular bone and MC of *Fgfr2^+/S252W^* embryos and their *Fgfr2^+/+^* littermates at E16.5 by laser capture microdissection (LCM) (Fig. 3A). Total RNA from the two specific tissues was isolated and used for RNA-seq analysis.

We identified 122 genes that were significantly differentially expressed in the mandibular bone of *Fgfr2^+/S252W^* embryos compared to their *Fgfr2^+/+^* littermates (Table S3). Sixty-seven differentially expressed genes (DEGs) were upregulated and 55 DEGs were downregulated in the mutant (Fig. 3B,C). Gene ontology (GO) analysis of DEGs in biological process (Fig. S6A) and cellular component (Fig. S6B) identified several GO terms including osteoclast differentiation (GO:0030316), bone remodeling (GO:0046849), bone resorption (GO:0045453), ossification (GO:0001503), and extracellular matrix (GO:0031012), all relevant to the histological phenotype of *Fgfr2^+/S252W^* embryonic mandibles. No significant DEGs were found in MC of *Fgfr2^+/S252W^* embryos compared to their *Fgfr2^+/+^* littermates (data not shown).

### Increased osteoclastogenesis in *Fgfr2^+/S252W^* hemimandibles

**Fig. 4.**
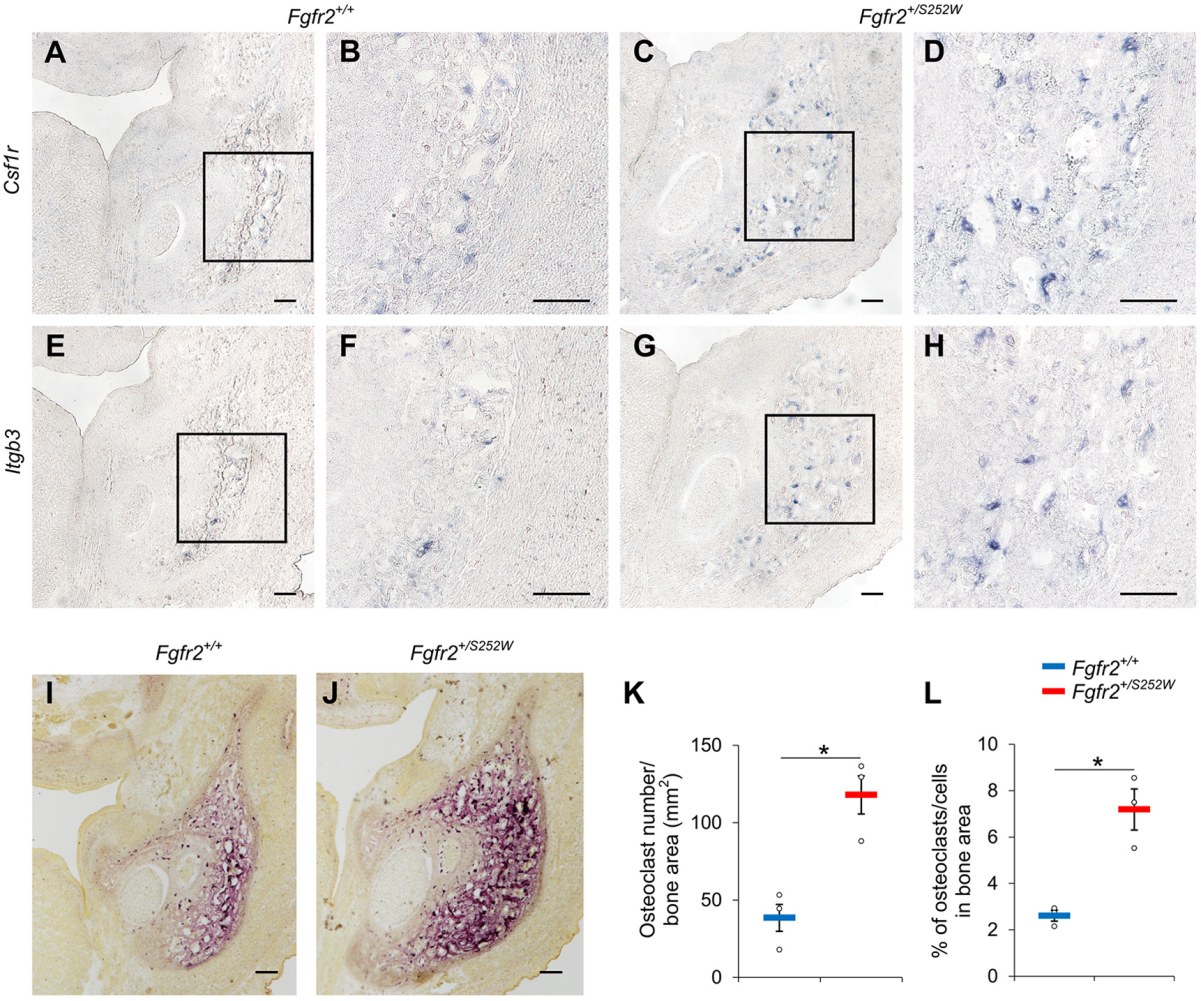
**Increased osteoclastogenesis in the mandibular bone of *Fgfr2^+/S252W^* embryos at E16.5.** (A-H) The differential expression of *Csf1r* and *Itgb3* in the mandible of *Fgfr2^+/+^* (A,B,E,F) and *Fgfr2^+/S252W^* (C,D,G,H) embryos were validated by *in situ* hybridization (ISH). B,D,F and H show higher magnification of the boxed areas in A,C,E and G, respectively. (I-J) TRAP assay stained osteoclasts (purple) in the mandible of *Fgfr2^+/+^* (I) and *Fgfr2^+/S252W^* (J) embryos. (K-L) Quantitative measurements of the density (K) and percentage (L) of osteoclasts in the bone area of *Fgfr2^+/+^* (*n*=3) and *Fgfr2^+/S252W^* (*n*=3) embryos. Data are mean±s.e.m. *P<0.05, two-tailed Welch's *t*-test. Scale bars: 100 µm.

A group of genes active in osteoclastogenesis, including *Acp5*, *Calcr*, *Csf1r*, *Ctsk*, *Il1r1*, *Itgb3*, *Oscar* and *Tnfrsf11a* (Table S3), were identified by transcriptome analysis as significantly upregulated in *Fgfr2^+/S252W^* embryos relative to their *Fgfr2^+/+^* littermates. To validate our sequencing analyses, we tested the expression of *Csfr1* and *Itgb3*, critical regulators of the osteoclast lineage, using section *in situ* hybridization (ISH) at E16.5. *Csf1r* encodes a tyrosine kinase growth factor that is the receptor for the ligand colony stimulating factor 1 (CSF1). CSF1R-mediated signaling plays an important role in osteoclastogenesis and *Csf1r^−/−^* mice exhibit severe osteoclast deficiency (Dai et al., 2002). Integrin beta 3, encoded by *Itgb3*, forms a complex with integrin alpha V, and integrin αvβ3 is essential for normal osteoclast function (McHugh et al., 2000). ISH showed that *Csf1r* exhibited a scattered expression pattern in the mandibular area of *Fgfr2^+/+^* littermates (Fig. 4A,B), labeling preosteoclasts and osteoclasts in the mandible. In the mandible of *Fgfr2^+/S252W^* embryos, increased *Csf1r-*positive cells were detected (Fig. 4C,D), consistent with the results of the transcriptome analysis. Similarly, there was increased expression of *Itgb3* in the mandible of *Fgfr2^+/S252W^* embryos (Fig. 4G,H) relative to their *Fgfr2^+/+^* littermates (Fig. 4E,F), indicating increased expression of osteoclast genes in mice carrying the FGFR2 S252W mutation. To confirm this result, we analyzed the tartrate-resistant acid phosphatase (TRAP) activity as a functional osteoclastic marker. TRAP staining revealed increased osteoclastic activity in the mandibular tissues of *Fgfr2^+/S252W^* embryos relative to *Fgfr2^+/+^* littermates at E16.5 (Fig. 4I,J). The number of osteoclasts per bone area in mutant embryos was significantly increased compared to *Fgfr2^+/+^* littermates (Fig. 4K, 38.5±8.7 osteoclasts/mm^2^ for *Fgfr2^+/+^*, 117.9±12.3 osteoclasts/mm^2^ for *Fgfr2^+/S252W^*, *P*=0.0158; data are mean±s.e.m.), and the percentage of osteoclasts in the bone area was significantly increased (Fig. 4L, 2.61±0.23% for *Fgfr2^+/+^*, 7.19±0.89% for *Fgfr2^+/S252W^*, *P*=0.0288), suggesting higher bone resorption activity in mice carrying the mutation relative to the controls.

To test whether increased osteoclastic activity in the mandible observed in the *Fgfr2^+/S252W^* mice was a common mechanism for mandibular dysmorphogenesis in the other FGFR2-related mouse models, we performed the TRAP assay (Fig. S7). The *Fgfr2^+/P253R^* embryos (Fig. S7C), like the *Fgfr2^+/S252W^* (Fig. 4J and Fig. S7B) embryos, showed increased osteoclastic activity compared with unaffected littermates (Fig. S7A). The *Fgfr2c^C342Y/+^* embryos showed increased staining in a smaller area of the mandible (Fig. S7E), which also exhibited impaired mineralization (Fig. S5H,L) compared to the unaffected littermates (Fig. S7D). The increase of osteoclastic activity was consistent to the relative magnitude of changes in mandibular morphology and histology in the three mouse models (*Fgfr2^+/S252W^*>*Fgfr2^+/P253R^*>*Fgfr2c^C342Y/+^*), indicating abnormal osteoclastogenesis in the mandible is an important process affecting the relative morphogenesis in FGFR2-related mouse models.

### Increased expression of *Enpp1* and *Ank*, and elevated inorganic pyrophosphate (PPi) concentration in *Fgfr2^+/S252W^* mandible

GO analysis (Fig. S6) showed that the expression of many genes that contribute to ossification (GO:0001503) were downregulated in the mandible of *Fgfr2^+/S252W^* embryos, including *Aspn*, *Chrdl1*, *Igf1*, *Mgp*, *Ptn*, *Sfrp2*, *Thbs3* and *Tnn*, suggesting that osteogenesis was inhibited in the *Fgfr2^+/S252W^* mandible. Mineralization plays a pivotal role in bone formation and is initiated within matrix vesicles (MVs), in which Ca^2+^ ions and inorganic phosphate (Pi) crystallize to form hydroxyapatite (HA). The extracellular PPi (ePPi) adsorbs tightly to HA and potently antagonizes the ability of Pi to crystallize with calcium to form HA, inhibiting HA crystal propagation (Terkeltaub, 2001). *Enpp1* and *Ank* are essential in regulating levels of PPi (Mackenzie et al., 2012). *Enpp1* is expressed in differentiated osteoblasts and encodes ectonucleotide pyrophosphatase/phosphodiesterase 1 (ENPP1) protein that influences matrix mineralization by increasing extracellular levels of PPi and regulates osteoblast differentiation (Nam et al., 2011). *Enpp1* is essential for normal bone development and control of physiological bone mineralization, and *Enpp1^−/−^* mice are characterized by severe disruption to the architecture and mineralization of long bones, dysregulation of calcium/phosphate homeostasis and changes in *Fgf23* expression (Mackenzie et al., 2012). *Ank* encodes the progressive ankylosis protein, which is a highly conserved transmembrane pyrophosphate transporter that channels PPi into the extracellular matrix (Chen et al., 2011). Mutations located in cytoplasmic domains close to the C terminus of the human homolog of the *Ank* gene (*ANKH*) were identified for the autosomal dominant form of craniometaphyseal dysplasia (CMD) (Nürnberg et al., 2001; Reichenberger et al., 2001). Overexpression of *Ank* in tissue culture cells leads to an increase in the total level of ePPi (Ho et al., 2000).

**Fig. 5.**
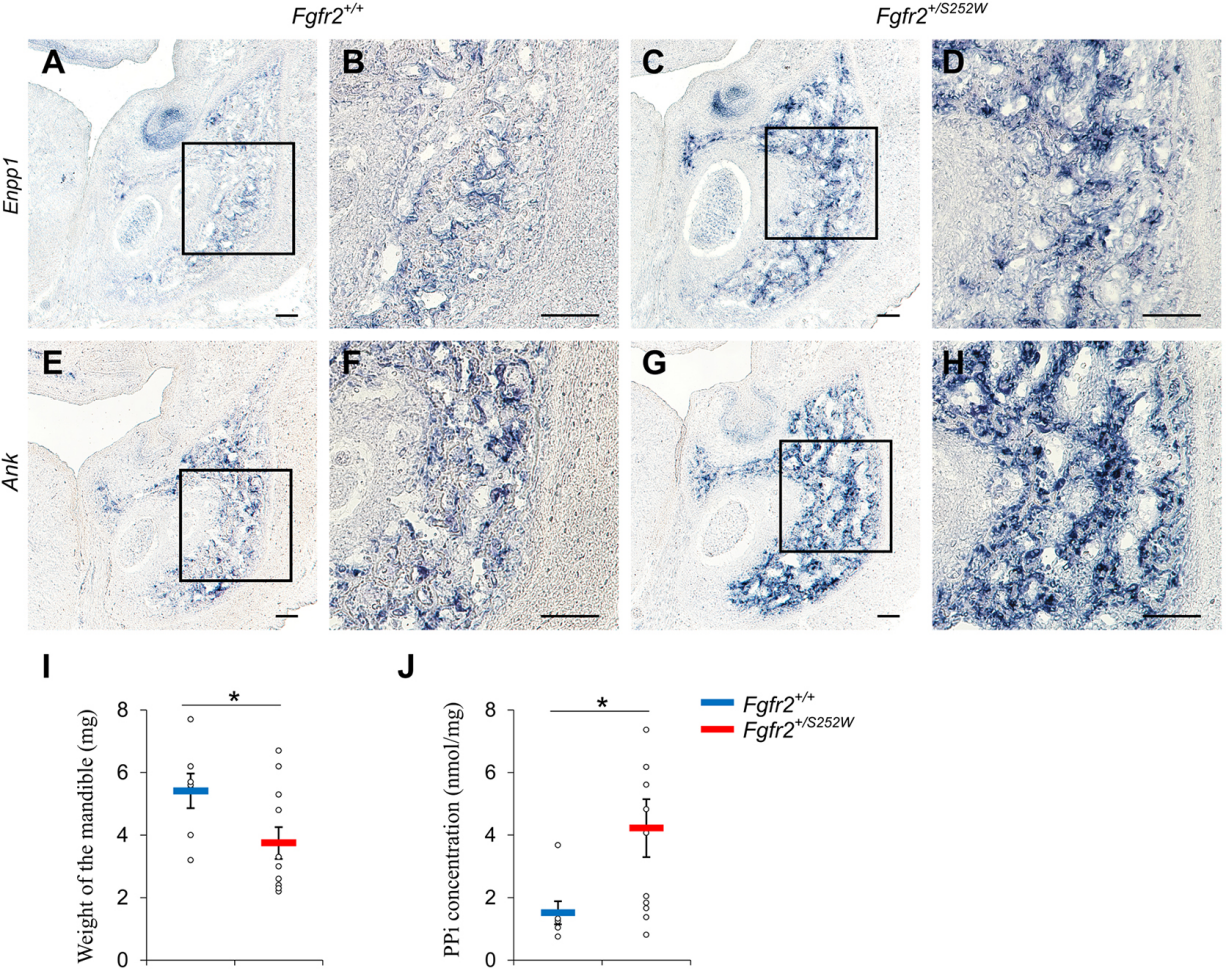
**Increased expression of *Enpp1* and *Ank* and elevated PPi concentration in the mandible of *Fgfr2^+/S252W^* embryos at E16.5.** (A-H) RNA expression of *Enpp1* and *Ank* in the mandible of *Fgfr2^+/+^* littermate (A,B,E,F) and *Fgfr2^+/S252W^* embryos (C,D,G,H) was validated using ISH. B,D,F and H show higher magnification of the boxed areas in A,C,E and G, respectively. The weight of the mandible (I) and PPi concentration in the mandible (J) were measured for *Fgfr2^+/+^* littermates (*n*=7) and *Fgfr2^+/S252W^* (*n*=11) embryos at E16.5. Data are mean±s.e.m. *P<0.05, two-tailed Welch's *t*-test. Scale bars: 100 µm.

*Enpp1* and *Ank* were among the most significantly upregulated genes in the mandible of *Fgfr2^+/S252W^* embryos at E16.5 (Fig. 3C), with log_2_ fold change of 1.83 and 1.33, respectively (Fig. 3C and Table S3). The differential expression was validated using ISH (Fig. 5A-H). To test whether upregulated expression of *Enpp1* and *Ank* was associated with elevated PPi levels, the E16.5 mandibles were dissected and the weight and amount of PPi were quantified (Fig. 5I,J). The weight of *Fgfr2^+/S252W^* mandibles (3.75±0.48 mg) was significantly lower than controls (5.41±0.51 mg), whereas PPi concentrations in the mutant embryonic mandibles were higher (4.22±0.93 nmol/mg) relative to those of the *Fgfr2^+/+^* littermates (1.52±0.37 nmol/mg). These findings suggest that genes that were transcriptionally dysregulated by the FGFR2 S252W mutation changed the Pi/PPi balance toward reduced bone formation and mineralization.

### Increased proliferation of osteoblasts and chondrocytes in the mandible of *Fgfr2^+/S252W^* embryos

**Fig. 6.**
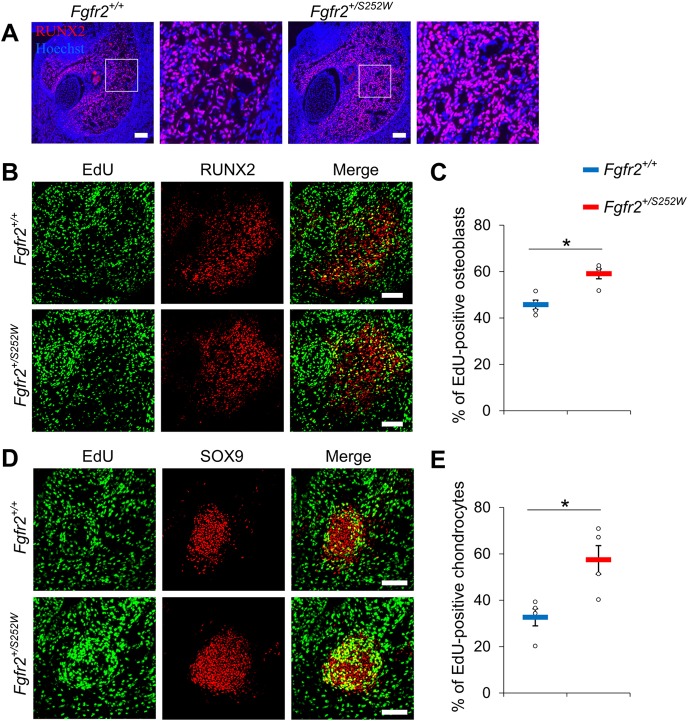
**Increased cell proliferation of osteoblasts and chondrocytes in the mandible of *Fgfr2^+/S252W^* embryos.** (A) The osteoblasts in the mandibular bone at E16.5 were visualized using IHC for RUNX2. Boxed areas are shown at higher magnification on the right, respectively. (B) Double staining with EdU assay (green) and IHC for RUNX2 (red) at E12.5. (C) The percentage of proliferating osteoblasts (EdU-positive) in the total osteoblasts (RUNX2-positive) is shown for *Fgfr2^+/+^* (*n*=4) and *Fgfr2^+/S252W^* (*n*=4) embryos. (D) EdU assay (green) with IHC for SOX9 (red) in MC at E12.5. (E) The percentage of proliferating cells (EdU-positive) in MC (SOX9-positive) is shown for *Fgfr2^+/+^* (*n*=4) and *Fgfr2^+/S252W^* (*n*=4) embryos. Data are mean±s.e.m. *P<0.05, two-tailed Welch's *t*-test. Scale bars: 100 µm.

The osteogenic tissue and MC of *Fgfr2^+/S252W^* embryos at E16.5 were enlarged relative to their littermates that did not carry the mutation (Fig. 2B). IHC for RUNX2 as an osteoblast marker confirmed that osteoblasts are increased in the osteogenic region of *Fgfr2^+/S252W^* embryos (Fig. 6A). However, there is no indication of this from RNA-seq analysis of mandibular bone or MC at E16.5, suggesting that the molecular changes resulting from the FGFR2 S252W mutation might have occurred at an earlier stage to cause the enlargement. As osteoblasts with the FGFR2 S252W mutation have an increased capacity for proliferation and differentiation *in vitro* (Holmes et al., 2009; Yang et al., 2008) and FGF signaling plays an important role in chondrocyte proliferation (Brewer et al., 2016), we hypothesized that the FGFR2 S252W mutation would increase cell proliferation during the earlier stages of mandible development, resulting in increased cell numbers and enlargement of the osteogenic tissue and MC. To test this, the EdU assay was performed at E12.5 when ALP, an early osteoblast marker, can be detected in the jaw (Funato et al., 2016) and adjacent mesenchymal cells are condensing to form MC (Parada and Chai, 2015). RUNX2 was used as a marker for osteoblasts (Fig. 6B), and chondrocytes in the MC were visualized by staining for SOX9 (Fig. 6D), which was strongly expressed in immature/proliferating chondrocytes (Leung et al., 2011). The percentage of proliferating cells (EdU-positive) in osteoblasts (SOX9-positive) of *Fgfr2^+/S252W^* embryos was 59.7±2.1%, significantly increased compared with 45.7±2.0% in *Fgfr2^+/+^* littermates (Fig. 6C). The percentage of proliferating cells (EdU-positive) in MC (SOX9-positive) of *Fgfr2^+/S252W^* embryos was 57.4±6.1%, significantly increased compared with 32.6±3.7% in *Fgfr2^+/+^* littermates (Fig. 6E).

As apoptosis is another possible mechanism affecting cell numbers, the TdT-mediated dUTP nick end labeling (TUNEL) assay was performed on the mandible of *Fgfr2^+/S252W^* and *Fgfr2^+/+^* embryos at E16.5. TUNEL signal was detected in the mandibular bone of both groups (Fig. S8A). No apoptotic chondrocytes in MC were observed but signals were detected in perichondrium (Fig. S8A), similar to a previous study on normal MC development (Amano et al., 2010). Quantitative analysis shows insignificant increase of TUNEL-positive cells in mandibular bone (Fig. S8B, 33.1±5.7 for *Fgfr2^+/+^* and 60.3±7.8 for *Fgfr2^+/S252W^*, *P*=0.089) and perichondrium (Fig. S8C, 6.8±0.6 for *Fgfr2^+/+^* and 9.0±0.5 for *Fgfr2^+/S252W^*, *P*=0.083) of *Fgfr2^+/S252W^* embryos compared with *Fgfr2^+/+^* littermates, indicating that apoptosis does not contribute to the enlargement of the osteogenic tissue or MC.

## DISCUSSION

The findings of this study demonstrate that the 3D dysmorphology of hemimandibles of three FGFR2-related mouse models for craniosynostosis syndromes are easily distinguished from the mandibles of their respective unaffected littermates at birth. Focusing on the hemimandibles of *Fgfr2^+/S252W^* mice, we demonstrated significant dysmorphology, increase in MC size and dysmorphic bony microarchitecture in embryos at E16.5. Our analyses showed that these changes are due in part to inhibited osteogenic activity with increased osteoclastogenesis of the mandible and earlier increased proliferation of osteoblasts and chondrocytes.

There are comparative data relevant to this study from previous analyses of humans and adult mice. Most studies of the mandible in humans with craniosynostosis syndromes have concluded that the mandible is intrinsically normal and that the morphological differences that are noted likely represent a developmental response to the composite of structural variations in the basicranium and midface in these conditions (Enlow, 2000; Wink et al., 2013). In humans, the data are necessarily postnatal and analyses of mandibular shape are often conducted after individuals have undergone reconstructive surgery of the midface. Because the maxilla and mandible function as a unit in phonation and mastication, surgically induced changes of midfacial morphology could affect mandibular growth and morphology through changes in functional relationships.

Previous mandibular analyses in mouse models for craniosynostosis include little or no quantitative or gene expression information for embryonic and newborn mice, although it has been reported that *Fgfr2* is expressed in mandibular osteoblasts (Rice et al., 2003). A recent study using only mandibular morphometry of adult mice carrying the FGFR2c C342Y mutation on a mixed genetic background (72% C57BL6/J and 28% Swiss) reported significantly reduced ascending (measured from the apex of coronoid process to gonion) and descending (measured from the apex of coronoid process to menton) mandibular heights, mandibular lengths (measured from condylion to pogonion), and intercoronoid and intercondylar widths, but increased intergonial widths (Khominsky et al., 2018). Though the method of measurement differs from ours, these observations generally agree with our data for newborn mice carrying the same mutation on a CD1 background. Mice aged 4 and 8 weeks carrying the FGFR2 P253R mutation causative for Apert syndrome display globally reduced mandibular dimensions (Du et al., 2010). Results presented here show that the mandibles of newborn *Fgfr2^+/P253R^* mice are generally reduced in size relative to their normal littermates at birth, and that the reduction is of greater magnitude in the posterior portion (including the posterior mandibular body, and coronoid, condylar and angular processes), contributing to a complex change in shape. We also reported that adult *Fgfr2^+/S252W^* Apert syndrome mice have a very small mandible with a dysmorphic angular process (Wang et al., 2005, 2010).

The molecular changes that result from FGFR mutations are complex and include constitutive (ligand-independent) or ligand-dependent FGFR activation, loss of function and altered cellular trafficking of receptors (Ornitz and Itoh, 2015). The FGFR2c C342Y mutation associated with Crouzon/Pfeiffer syndrome lies within the Ig-III domain of FGFR2c, and results in constitutive activation of the receptor (Mangasarian et al., 1997). FGFR2 S252W and P253R mutations are in the linker region, resulting in increased ligand affinity and altered specificity (Andersen et al., 1998; Ibrahimi et al., 2001; Yu et al., 2000). Crystal structures of the FGFR2 S252W and P253R mutations indicate that P253R indiscriminately increases the affinity of FGFR2 toward any FGF, whereas the S252W mutation selectively enhances the affinity of FGFR2 toward a limited subset of FGFs (Ibrahimi et al., 2001). These mutations in FGFR2 then differentially affect FGFR2 intracellular signaling pathways (e.g. ERK1/2, PLCγ/PKCα and PI3K/Akt), resulting in alterations in cell proliferation, differentiation and apoptosis, depending on the stage of cell differentiation (Ornitz and Marie, 2015), forming the basis for different mandibular phenotypes.

Zhou et al. demonstrated a significant decrease in mandibular cortical bone, decreased bone mass, a significant decrease in calcein labeling of mineralizing surfaces, and a reduced mineral apposition rate in the postnatal mandibles of *Fgfr2^S252W/+^* mice at P28 and P56 (Zhou et al., 2013). An observed increase in the number of osteoclasts, and a decreased number of osteoblasts per bone surface area, suggested lower bone formation capacities in *Fgfr2^S252W/+^* adult mandibles relative to those of *Fgfr2^+/+^* littermates. Bone modeling increases bone mass, changes the shape of bones and occurs throughout life, whereas bone remodeling functions to renew bone (Allen and Burr, 2015). If the cellular activities reported in the mandibles of adult *Fgfr2^S252W/+^* mice (Zhou et al., 2013) are functioning primarily to renew bone, although our results reflect changes in cellular activities that increase bone mass and change its shape, then the balance of the amount of tissue resorbed and formed at any particular site may be disrupted by the effect of FGFR2 S252W in modeling and remodeling.

Our results demonstrate an intrinsic difference in mandibular morphology of newborn mice carrying FGFR2-related craniosynostosis mutations. We used ALP staining to detect mature osteoprogenitor cells, preosteoblasts and differentiated osteoblasts (Huang et al., 2007), and found that cell numbers in ALP-positive areas were increased in *Fgfr2^+/S252W^* embryos, indicating that the FGFR2 S252W mutation promotes osteoblastic proliferation and differentiation, consistent with results of previous analyses of adult cranial bone response to FGFR2 mutations (Holmes et al., 2009; Yang et al., 2008). However, the overall mandible is reduced in size in mutant mice both pre- and postnatally. Our finding of increased osteoclastogenesis is a mechanism that can account for the overall reduction in size of mandibles of mice carrying FGFR2 mutations.

Investigation of the transcriptome of the mandible in embryonic mice carrying the FGFR2 S252W mutation revealed dysregulation of genes involved in bone formation, bone mineralization and osteoclastogenesis, highlighting increased expression of genes undergoing osteoclast differentiation and dysregulated genes active in bone mineralization. Bone formation and bone resorption are important determinants of bone size and shape, whether osteoblast and osteoclastic activity are coupled (as in remodeling) or uncoupled (as in modeling) (Allen and Burr, 2015). Altered signaling pathways result in the dysregulation of genes that are involved in osteoclastogenesis, bone formation and bone mineralization, contributing to impaired mandibular morphogenesis and microarchitecture. During normal growth, as bone mass increases, resorption is required to alter bone shape and maintain a functioning skeletal element. Our data show that improper regulation of osteoblastogenesis and osteoclastogenesis can offset the balance required for bone modeling contributing to changes in the developmental trajectory of individual embryonic bones and resulting in altered bone phenotypes. The FGFR2 S252W mutation may impair mandibular bone formation and contribute to compromised skeletal architecture by regulating both osteoblastogenesis and osteoclastogenesis.

MC plays a crucial role as a supportive tissue for mandible formation and early growth. Chondrocytes that form endochondral bone are differentiated from mesodermal cells in general, whereas cells forming MC are differentiated from cells of neural crest origin (Amano et al., 2010). In addition, the boundary between neural crest and mesoderm cells of the chondrocranium lies between the hypophyseal and parachordal cartilages (McBratney-Owen et al., 2008), indicating that the cartilages caudal to the hypophyseal cartilage are of mesodermal origin. Abnormal MC development is associated with dysmorphogenesis of the mandible. For example, activating FGFR3 mutations associated with achondroplasia lead to structural anomalies of MC and condylar cartilages of the mandible, resulting in mandibular hypoplasia and dysmorphogenesis (Duplan et al., 2016). We observed dramatically increased size and cell number in MC of *Fgfr2^+/S252W^* embryos, with increased proliferation of chondrocytes detected as early as E12.5. Previous studies have shown that *Fgfr2^+/S252W^* mutant mice have increased cartilage of the basicranium (posterior to the hypophyseal cartilage) and thickened nasal cartilage owing to increased chondrocyte proliferation (Wang et al., 2005; Holmes et al., 2018). These results suggest a common mechanism of increased proliferation by the FGFR2 S252W mutation in these cranial cartilages, whether derived from cranial neural crest cells or mesoderm.

**Fig. 7.**
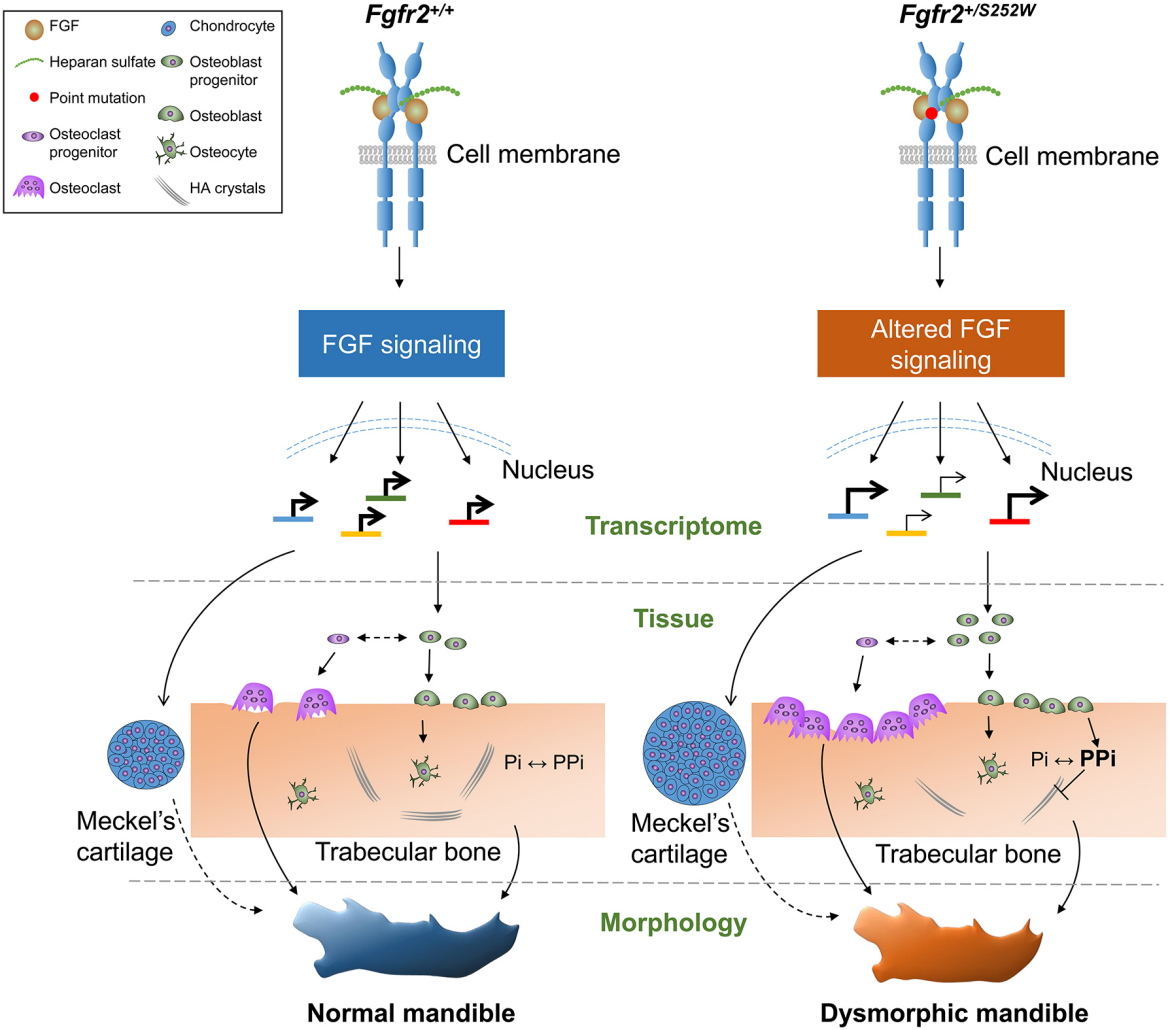
**Proposed model of mandibular dysmorphogenesis in prenatal development of *Fgfr2^+/S252W^* mice.** In the mandible of *Fgfr2^+/+^* mice, FGFR2 signaling is activated by specific FGF binding, forming a complex of FGFs, heparan sulfate and FGFRs. The linker region between the immunoglobulin-like domains II and III regulates the ligand binding specificity and affinity. Dimerization and transphosphorylation by kinases in the intracellular domain of FGFR2 cause activation of downstream signaling cascades. These activated signaling pathways can regulate gene expression, controlling osteogenesis, osteoclastogenesis and chondrogenesis. Transcriptional level: The FGFR2 S252W mutation alters ligand specificity and affinity, resulting in abnormal FGFR2 signaling, which dysregulates the transcriptome in different cell types. Tissue level: Osteoblast proliferation is activated, enlarging the osteogenic tissue. Increased PPi inhibits mineralization, and bone resorption is promoted through osteoclastic activity, causing a change in the microarchitecture. Meckel’s cartilage is affected by increased proliferation of chondrocytes, resulting in an increase in size. Morphological level: Abnormal osteogenic activities contribute to changes in mandibular shape.

In summary, we quantitatively analyzed prenatal mandibular morphology in mouse models carrying mutation variants of *Fgfr2* that are associated with craniosynostosis syndromes when present in humans. Finding that the mandibles of *Fgfr2^+/S252W^* mice were quantitatively the most different from their unaffected littermates, we further studied the mandibles of these mice using histology, immunochemistry and transcriptome analyses to understand the source of altered morphology and abnormal microarchitecture of these mice with altered ligand affinity and specificity of FGFR2 (Cunningham et al., 2007). We have previously shown that the craniofacial phenotype (not including the mandible) of mice carrying the mutation in each Fgfr2 model is different compared to that of littermates not carrying the mutation (Martínez-Abadías et al., 2013; Motch Perrine et al., 2014; Wang et al., 2005, 2010). We suggest a model that mutation-induced changes in activated FGF signaling and downstream pathways are associated with dysregulation of osteoblastogenesis, osteoclastogenesis, resorption, mineralization and the formation of MC, resulting in dysmorphogenesis of the mandible (Fig. 7). Mandibular development is directly affected by the FGFR2 mutations in these mouse mutants, as was first suggested morphologically and later shown histologically by our results. Mandibular dysmorphogenesis in these mouse models for craniosynostosis results, at least in part, from the intrinsic effects of the mutation, and are not solely related to the functional relationship of the mandible with the midface and cranial base as previously deduced from human data.

## MATERIALS AND METHODS

### Mouse models

The generation of *Fgfr2^+/S252W^*, *Fgfr2^+/P253R^* and *Fgfr2c^C342Y/+^* models was previously described (Eswarakumar et al., 2004; Wang et al., 2005, 2010). *Fgfr2^+/S252W^* and *Fgfr2^+/P253R^* models were maintained on a C57BL6/J background. The *Fgfr2c^C342Y/+^* model was maintained on a CD1 background for viability and breeding. Our samples for µCT analyses consisted of 182 newborn (P0) mice [*Fgfr2^+/S252W^* model: 22 mutants (9 females, F:13 males, M); 24 unaffected littermates (20 F:4 M); *Fgfr2^+/P253R^* model: 35 mutants (20 F:15 M); 28 unaffected littermates (16 F:12 M); *Fgfr2c^C342Y/+^* model: 34 mutants (18 F:16 M); 39 unaffected littermates (24 F:15 M)]. P0 mice were euthanized by inhalation anesthetics and fixed in 4% paraformaldehyde. Gestation time was 19.0±0.5 days. Samples for histological and transcriptome analysis consisted of 91 embryos [*Fgfr2^+/S252W^* model: 29 mutants (17 F:12 M); 37 unaffected littermates (21 F:16 M); *Fgfr2^+/P253R^* model: 8 mutants (2 F:6 M); 5 unaffected littermates (3 F:2 M); *Fgfr2c^C342Y/+^* model: 8 mutants (4 F:4 M); 4 unaffected littermates (3 F:1 M)]. Genotyping of tail DNA using PCR was performed to distinguish mutants and unaffected littermates. Mouse litters were produced in compliance with animal welfare guidelines approved by Icahn School of Medicine at Mount Sinai and Pennsylvania State University Animal Care and Use Committees.

### Imaging protocols

High-resolution µCT images with voxel size ranging from 0.014 to 0.025 mm were acquired by the Center for Quantitative X-Ray Imaging at the Pennsylvania State University (www.cqi.psu.edu) using the HD-600 OMNI-X high-resolution X-ray computed tomography system (Bio-Imaging Research Inc.). A minimum threshold of 70-100 mg/cm^3^ partial density HA (based on HA phantoms imaged with specimens) was used to reconstruct isosurfaces in Avizo 6.3 (Visualization Sciences Group). 3D coordinates of 32 biologically relevant landmarks (Fig. S1 and Table S1) were collected from the isosurfaces. Specimens were digitized twice, and measurement error was minimized by averaging coordinates of the two trials (maximum accepted error in landmark placement=0.05 mm).

### Statistical evaluation of shape differences

Variation in mandible shape was assessed by PCA using SAS 9.4 (SAS Institute). PCA summarizes the variation of large numbers of variables into a lower-dimensional space defined by principal component (PC) axes that are mutually orthogonal linear combinations of the linear distance data. The scores of an observation (mandible or mandibular region) on the PC axes map that observation into the space. We performed PCA (Darroch and Mosimann, 1985; Falsetti et al., 1993) of form (size and shape together) using inter-landmark linear distances estimated using the full mandible landmark set and subsets defining the left hemimandible (results of PCA analyses of left and right hemimandibles were similar), anterior body and ramus. Inter-landmark distances were *ln*-transformed, and their variance-covariance matrix was used as the basis for the PCA.

EDMA was used to statistically evaluate mandibular shape differences by hypothesis test and confidence interval estimation (Lele and Richtsmeier, 2001). EDMA is a 3D morphometric technique that is invariant to the group of transformations including translation, rotation and reflection (Lele and Richtsmeier, 1995; Richtsmeier and Lele, 1993). Briefly, the original 3D coordinates of landmark locations are rewritten and analyzed as a matrix of all unique linear distances among landmarks called the form matrix (FM). An average FM is estimated for each sample (Lele and Richtsmeier, 1995). The difference between samples is evaluated by estimating ratios of like-linear distances using sample-specific average FMs. The resulting matrix of ratios, the form difference matrix (FDM), is a collection of relative differences among landmarks used to define the forms. A non-parametric bootstrap procedure (100,000 resamples) is used to obtain confidence intervals for elements (each corresponding to a linear distance) of the FDM (Lele and Richtsmeier, 2001) that reveals the localized effects of the mutations on the mandible. We also include a non-parametric bootstrap assessment of the null hypothesis that the mean forms of two samples are the same (Lele and Richtsmeier, 2001). We tested for form difference of the entire left hemimandible, the anterior body and the ramus portion using WinEDMA (Cole, 2002).

### Bone volume, surface area and bone mineral density analyses

Bone volumes and surface areas were determined using the high-resolution µCT scans described above using Avizo 9.4 (Thermo Fisher Scientific). The minimum thresholds used to create isosurfaces ranged from 70-100 mg/cm^3^ partial density HA. The isosurfaces were then analyzed using the Material Statistics module of Avizo 9.4 software to determine bone volumes and bone surface area. Stradwin v5.3 (http://mi.eng.cam.ac.uk/~rwp/stradwin) was used to create isosurfaces from 30 of the hemimandibles (five of each group) (Treece et al., 2010, 2012). The tooth was excluded manually by placing guiding contours every five tomographic slices along the hemimandibles. Density values were determined from the partial density HA phantom normalized gray values at each isosurface vertex (∼89,000-112,000 measurements). Isosurfaces and their associated density values were registered using wxRegSurf v17 (http://mi.eng.cam.ac.uk/~ahg/wxRegSurf) (Gee et al., 2015; Stephens et al., 2018). A statistical shape model (SSM) was generated for the full dataset and for each mouse model. Mean density was calculated for each corresponding vertex and mapped onto the pertinent SSM. Pairwise statistical differences between littermates were determined by performing a linear model comparison at each vertex of the full SSM in R, with the resulting *P*-values being mapped for visual comparison.

### Histological analysis

Mouse embryos at E16.5 were dissected and the heads were fixed in 4% paraformaldehyde overnight at 4°C and then washed 3× with PBS. Samples were infused in 0.5 M sucrose in PBS until tissue sank, and then quick-frozen in optimal cutting temperature compound (OCT). Samples were sectioned at a thickness of 10 μm. For ALP and Alcian Blue staining, cryosection samples were incubated for 5 min at room temperature (RT) in 100 mM Tris-maleate buffer (pH 9.2) and then incubated for 5 min at RT in freshly prepared ALP substrate solution [100 mM Tris-maleate buffer (pH 9.2), 0.2 mg/ml naphthol AS-MX phosphate and 0.4 mg/ml Fast Red TR]. The slides were washed briefly and then stained with Alcian Blue solution [1% Alcian Blue, 3% acetic acid (pH 2.5)] for 5 min at RT. The slides were washed with water and then stained with 1 µg/ml Hoechst 33258 (Invitrogen Life Technologies) in PBS for 5 min. Images were collected in brightfield for ALP and Alcian Blue staining and then in UV for Hoechst 33258-stained nuclei. Calcium deposits were detected with Alizarin Red S staining solution (MilliporeSigma). Presence of phosphate was detected with von Kossa staining kit (American MasterTech Scientific). TRAP staining was performed using cryosections with Acid Phosphatase, Leukocyte (TRAP) Kit (MilliporeSigma) following the manufacturer's instructions. Images were analyzed using ImageJ for stained particles and areas.

### LCM and RNA-seq

LCM was performed as previously described in detail (Holmes et al., 2018). The heads of female *Fgfr2^+/+^* littermates (*n*=3) and female Apert *Fgfr2^+/S252W^* (*n*=3) embryos at E16.5 were embedded in OCT without fixation and rapidly frozen. Coronal cryosection was performed for the mandible at 12 µm thickness. The mandibular tissue and MC were captured and collected, respectively. RNA was isolated using an Arcturus Picopure RNA Isolation Kit (Thermo Fisher Scientific). Library preparation with NuGEN Ovation RNA-seq System v2 (NuGEN Technologies) and Nextera XT Library Prep kit (Illumina) was performed by the Gene Expression Core Facility at the Cincinnati Children's Hospital Medical Center as described (Holmes et al., 2018). Library sequencing was performed on an Illumina HiSeq 2500 instrument using standard protocols for paired-end 100 bp sequencing by the Genetic Resources Core Facility at the Johns Hopkins School of Medicine.

### Differential gene expression analysis and GO enrichment analyses

RNA-seq data processing, differential gene expression analysis and GO enrichment analyses were carried out as previously described (Holmes et al., 2018). Briefly, paired-end reads were mapped to the mouse (mm10) reference genome using STAR (Dobin et al., 2013) and gene count summaries were generated using featureCounts (Liao et al., 2014). Only genes with expression levels above 1 FPKM in at least 50% of samples were retained for further analysis. Normalization factors were computed on the filtered data matrix using the weighted trimmed mean of M-values method (Robinson and Oshlack, 2010), followed by voom mean-variance transformation in preparation for Limma linear modeling (Law et al., 2014). Data were fitted to a design matrix containing all sample groups, and pairwise comparisons were performed between sample groups. Finally, eBayes adjusted *P*-values were corrected for multiple testing using the Benjamini–Hochberg method and used to select genes with significant expression differences (*q*<0.05). For GO enrichment analyses, the ‘elim’ algorithm and ‘Fisher exact’ test were used to identify statistically over-represented GO categories at an FDR corrected *P*-value threshold of 0.05.

### RNA ISH

Differential gene expression identified by RNA-seq was validated by RNA ISH. Riboprobe templates were generated by PCR using primers from published literature or designed by Primer3 (http://primer3.ut.ee and Table S4), using cDNA derived from mouse embryonic total RNA at E11.5. Riboprobes were prepared with DIG RNA Labeling Mix (Roche Applied Science) as described by the manufacturer. RNA ISH was performed as previously described (Xu and Wilkinson, 1999).

### Quantification of PPi levels

Mandibles were isolated from embryos at E16.5 in cold PBS and dried briefly on delicate task wipers before the weight was measured. Quantification of PPi in the mandible was performed as previously described (Murali et al., 2016) with modification. Briefly, each mandible was incubated in 100 µl of 1.2 M HCl at 4°C overnight, neutralized with NaOH and diluted with water. Extracted PPi was quantified using the PPiLight Inorganic Pyrophosphate Assay (Lonza) according to the manufacturer's protocol.

### EdU assay and immunohistochemistry

EdU *in vivo* labeling was performed using single intraperitoneal injections of EdU to pregnant mice at E12.5 at a dose of 50 mg/kg body weight in a solution of 10 mg/ml PBS (pH 7.35) (Chehrehasa et al., 2009). The dams were sacrificed 30 min after the injection and embryos were dissected for cryosection. The heads were sectioned at a thickness of 10 μm and EdU-labeled cells were detected with Click-iT™ EdU Alexa Fluor™ 488 Imaging Kit (Thermo Fisher Scientific) followed by immunostaining for SOX9 (1:500, AB5535, MilliporeSigma) or RUNX2 (1: 200, HPA022040, MilliporeSigma) and Hoechst 33258 staining.

### TUNEL assay

TUNEL staining was performed using the *In Situ* Cell Death Detection Kit, Fluorescein (MilliporeSigma) according to the manufacturer's protocol.

## Supplementary Material

Supplementary information
