# Supplementary material for: Mandibular dysmorphology due to abnormal embryonic osteogenesis in FGFR2-related craniosynostosis mice
Source: Dis Model Mech. 2019 May 30;12(5):dmm038513. doi: 10.1242/dmm.038513 (PMC6550049; doi:10.1242/dmm.038513)
Supplement: Supplementary information [file dmm-12-038513-s1.pdf]

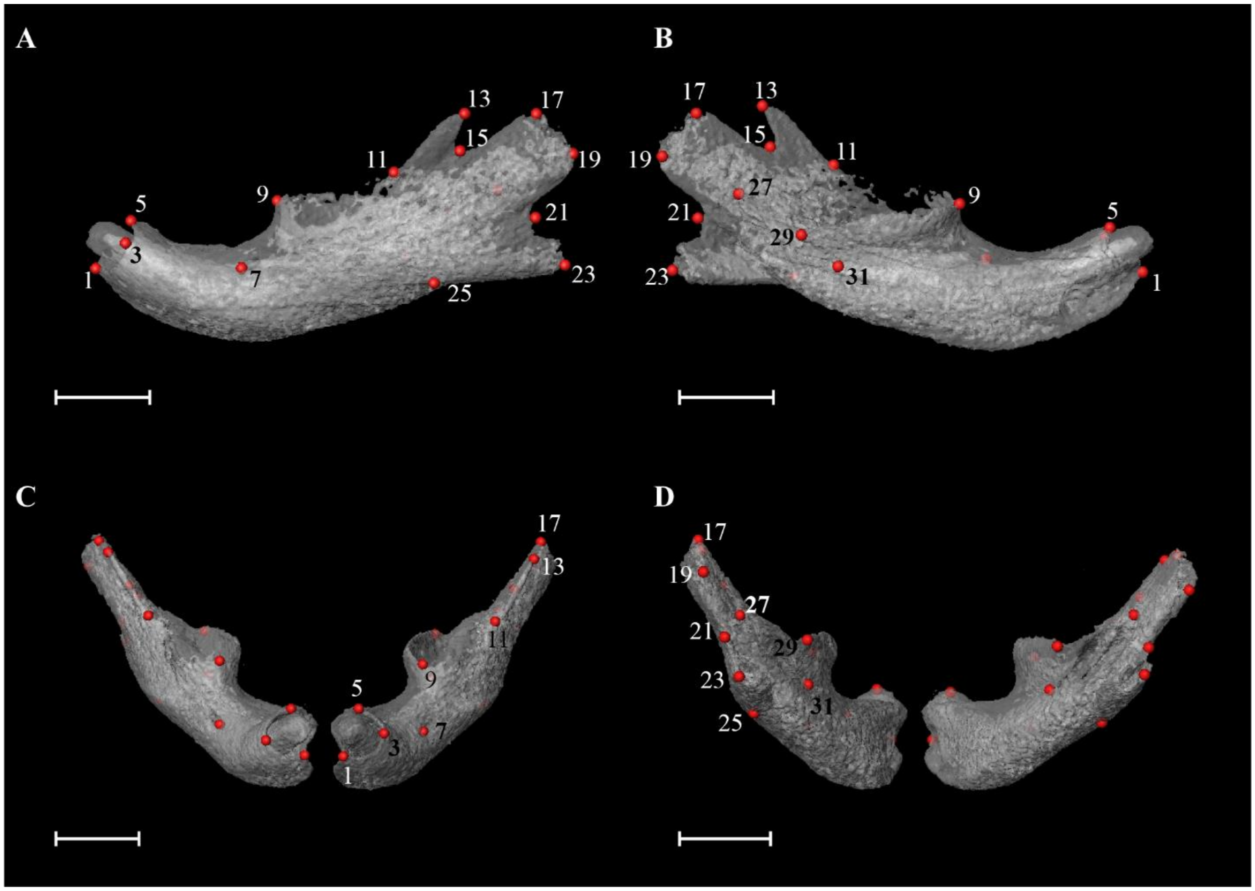

Fig. S1. Thirty-two anatomical landmarks located on left and right hemimandibles of newborn (P0) mice. Bilateral landmarks are annotated only on the left hemimandible. Landmark definitions are listed in Table S1. 3D coordinates of landmarks were collected from the buccal view (A) and lingual (B) view of an isosurface of the left hemimandible. Landmarks shown in the rostral (C) and caudal (D) views of the left and right hemimandibles are noted. Scale bar=1 mm.

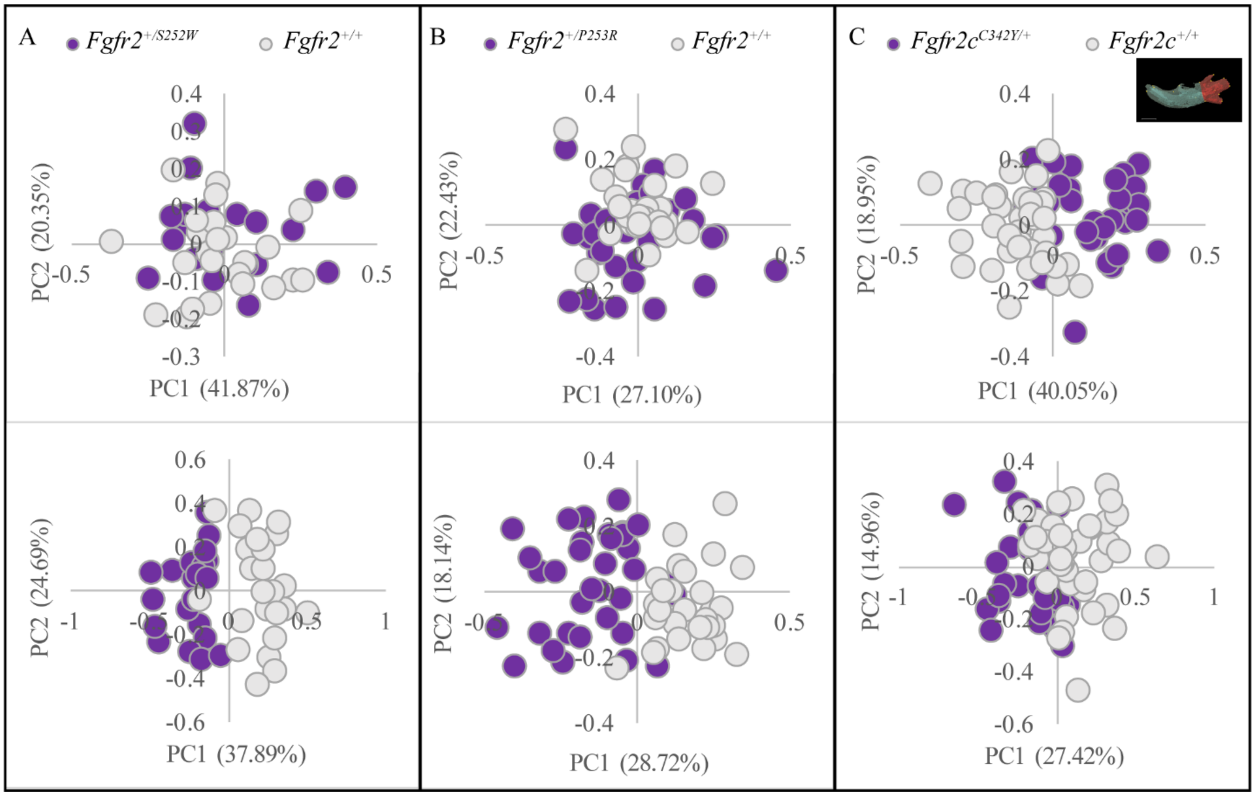

Fig. S2. Principal component analysis (PCA) of the anterior body and ramus portion of hemimandibles of three mouse models of craniosynostosis. Scatter plots of individual scores based on PCA from left hemimandibles of mutant and unaffected littermates of *Fgfr2*$^{+/S252W}$ (A) and *Fgfr2*$^{+/P253R}$ (B) Apert syndrome mouse models; and *Fgfr2c*$^{C342Y/+}$ (C) Crouzon/Pfeiffer syndrome mouse model along first and second Principal Components axes (PC1 and PC2). Separate PCAs were estimated for the anterior portion (anterior body, shown in blue in inset image) and posterior portion (ramus, shown in red in inset image) of the hemimandibles. PCA of the anterior body of the left hemimandible is shown in the top panel of each column while PCA of the left ramus portion is shown in the bottom panel.

Disease Models & Mechanisms • Supplementary information

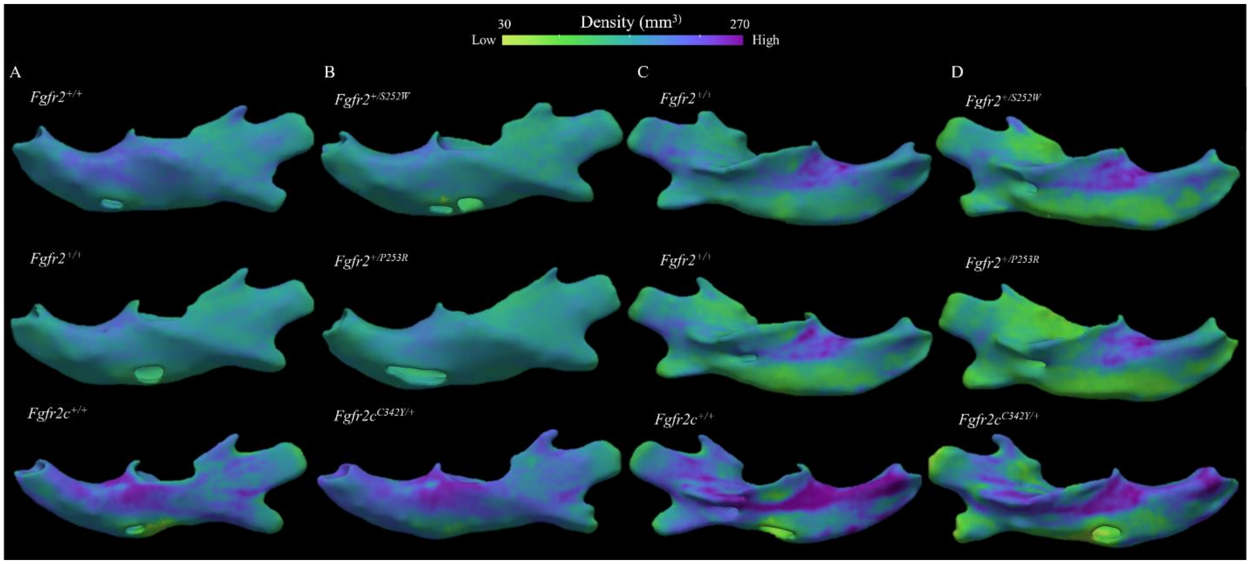

Fig. S3. Bone mineral density mapping. Bone mineral density (BMD) was mapped for each of the genotypes of interest: (A, B) buccal view of left hemimandibles of mice carrying *Fgfr2* mutations (B) and their respective unaffected littermates (A); (C, D) lingual view of left hemimandibles of mice carrying *Fgfr2* mutations (D) and their respective unaffected littermates (C). Though there are differences across the models, the contrast between mutant and respective unaffected littermates is greatest in the *Fgfr2*$^{+/S252W}$ mice.

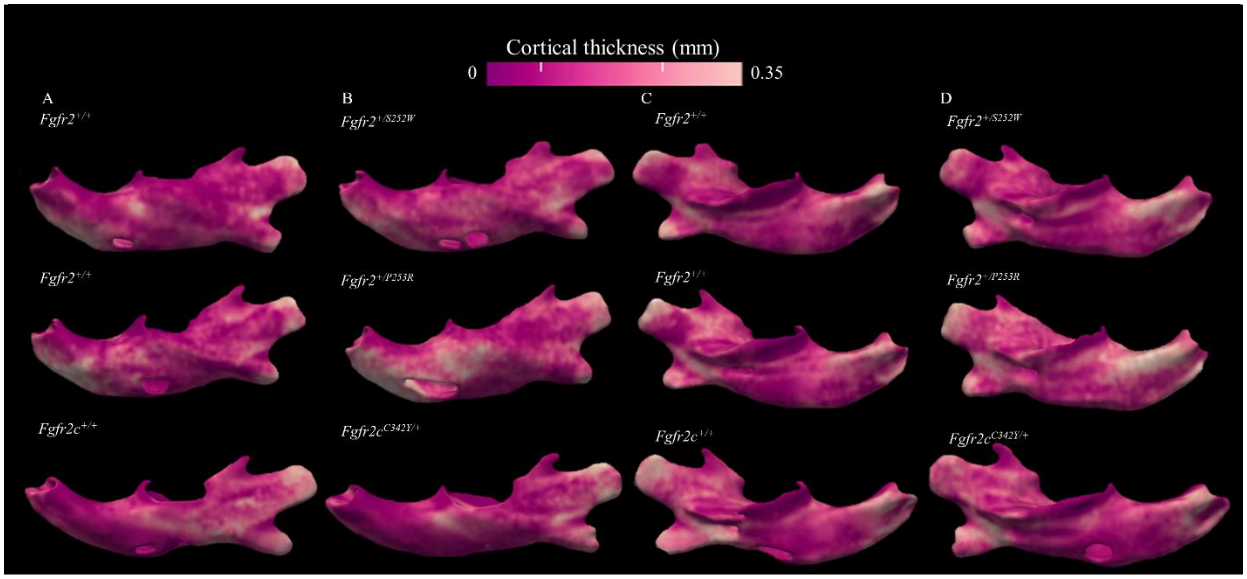

Fig. S4. Cortical bone thickness mapping. Cortical bone thickness was mapped for each of the genotypes of interest: (A, B) buccal view of left hemimandibles of mice carrying *Fgfr2* mutations (B) and their respective unaffected littermates (A); (C, D) lingual view of left hemimandibles of mice carrying *Fgfr2* mutations (D) and their respective unaffected littermates (C).

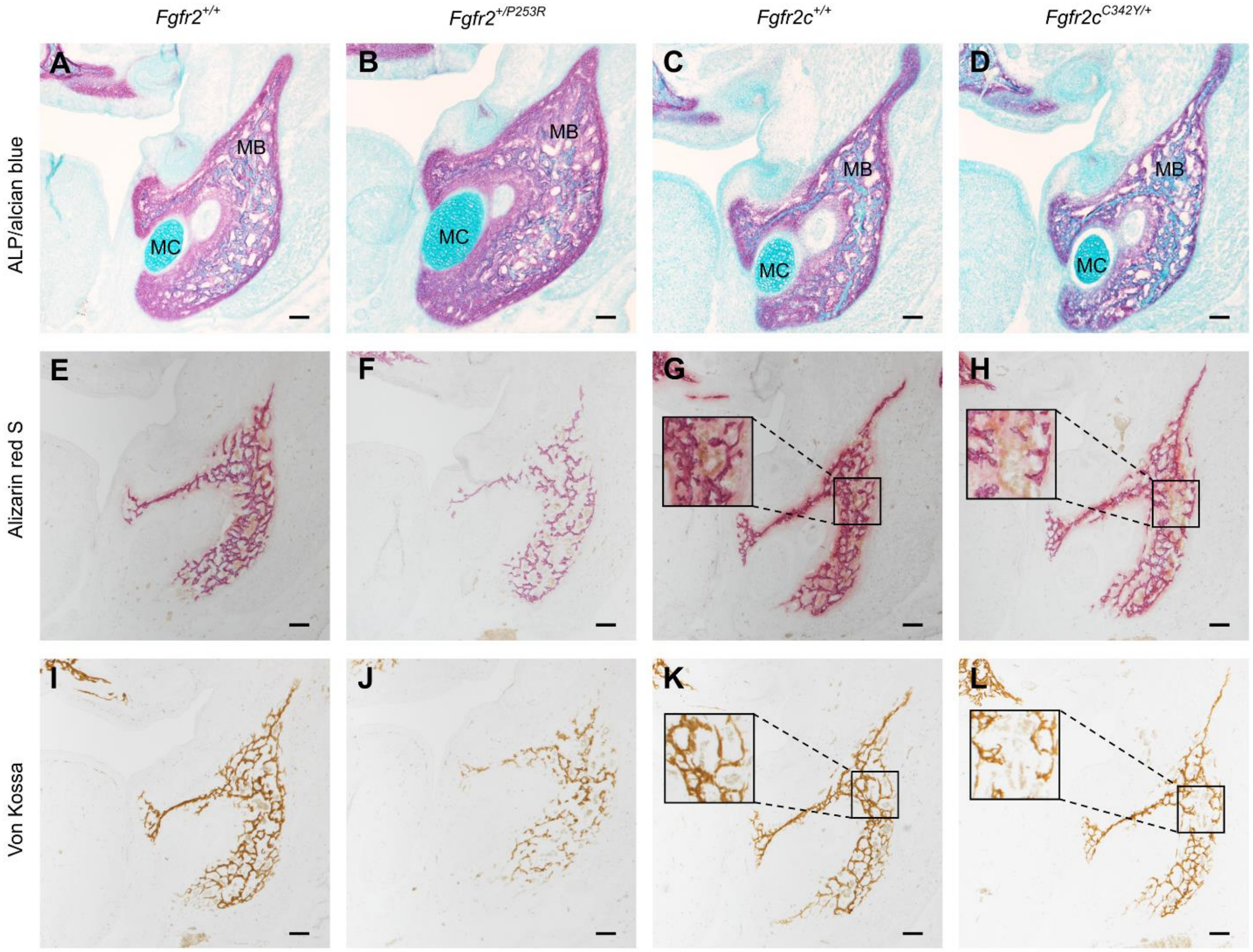

Fig. S5. Histological analysis of the mandibles of *Fgfr2*$^{+/P253R}$ and *Fgfr2c*$^{C342Y/+}$ embryos at E16.5. (A-D) ALP and alcian blue staining showed osteogenic tissues (red) and Meckel's cartilage (blue) in the mandible of *Fgfr2*$^{+/P253R}$ (B), *Fgfr2c*$^{C342Y/+}$ (D) and their *Fgfr2*$^{+/+}$ littermate embryos (A, C, respectively). MB, mandibular bone. MC, Meckel's cartilage. (E-H) Alizarin red S staining and (I-L) von Kossa staining showed ossification in the mandible of *Fgfr2*$^{+/P253R}$ (F, J) and *Fgfr2*$^{+/+}$ littermates (E, I), and *Fgfr2c*$^{C342Y/+}$ (H, L) and *Fgfr2*$^{+/+}$ littermates (G, K). Scale bar=100 µm. Boxes in G, H, K and L indicate the small area that exhibits impaired microarchitecture at a higher magnification in *Fgfr2c*$^{C342Y/+}$ embryos compared with *Fgfr2*$^{+/+}$ littermates.

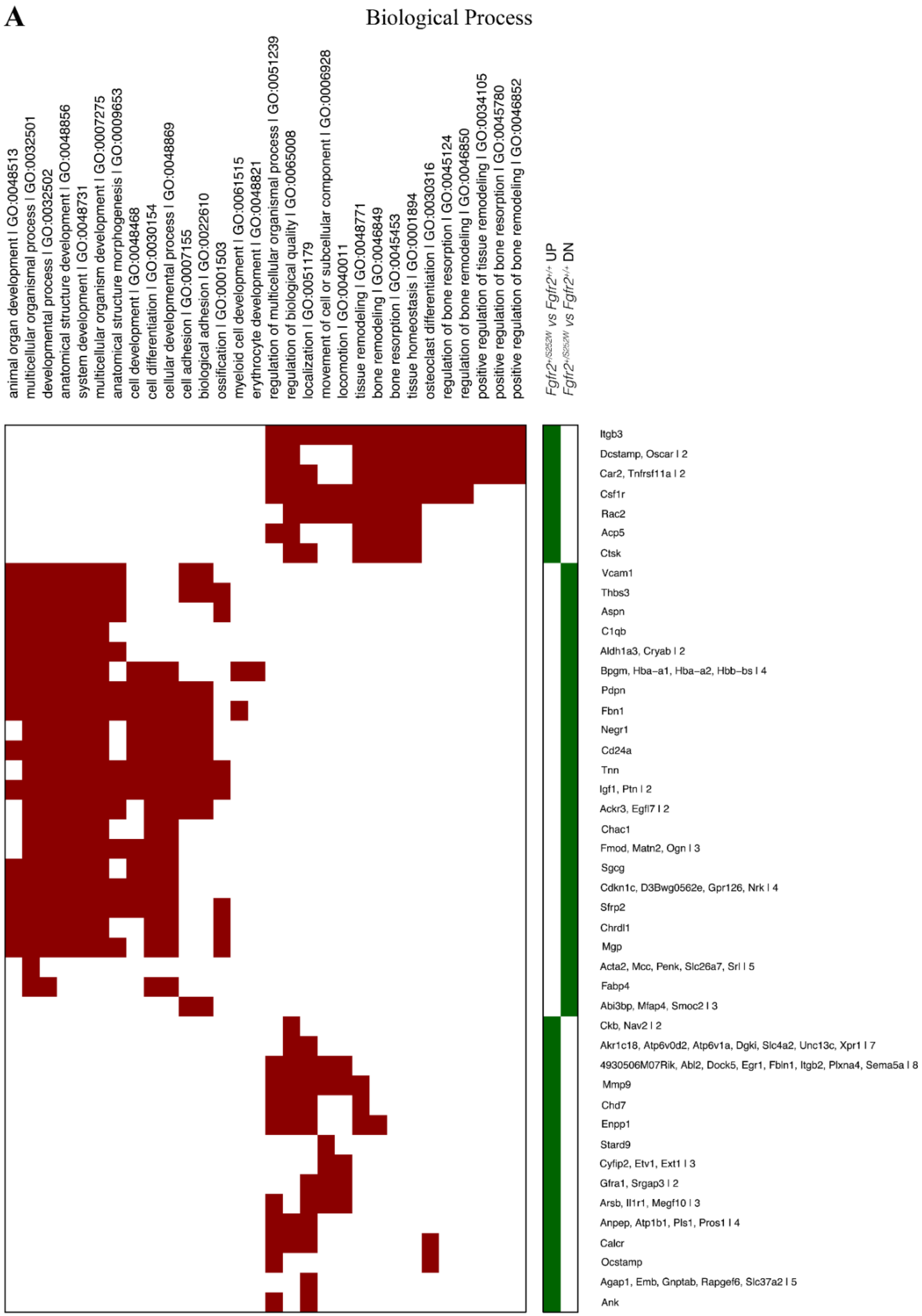

Disease Models & Mechanisms • Supplementary information

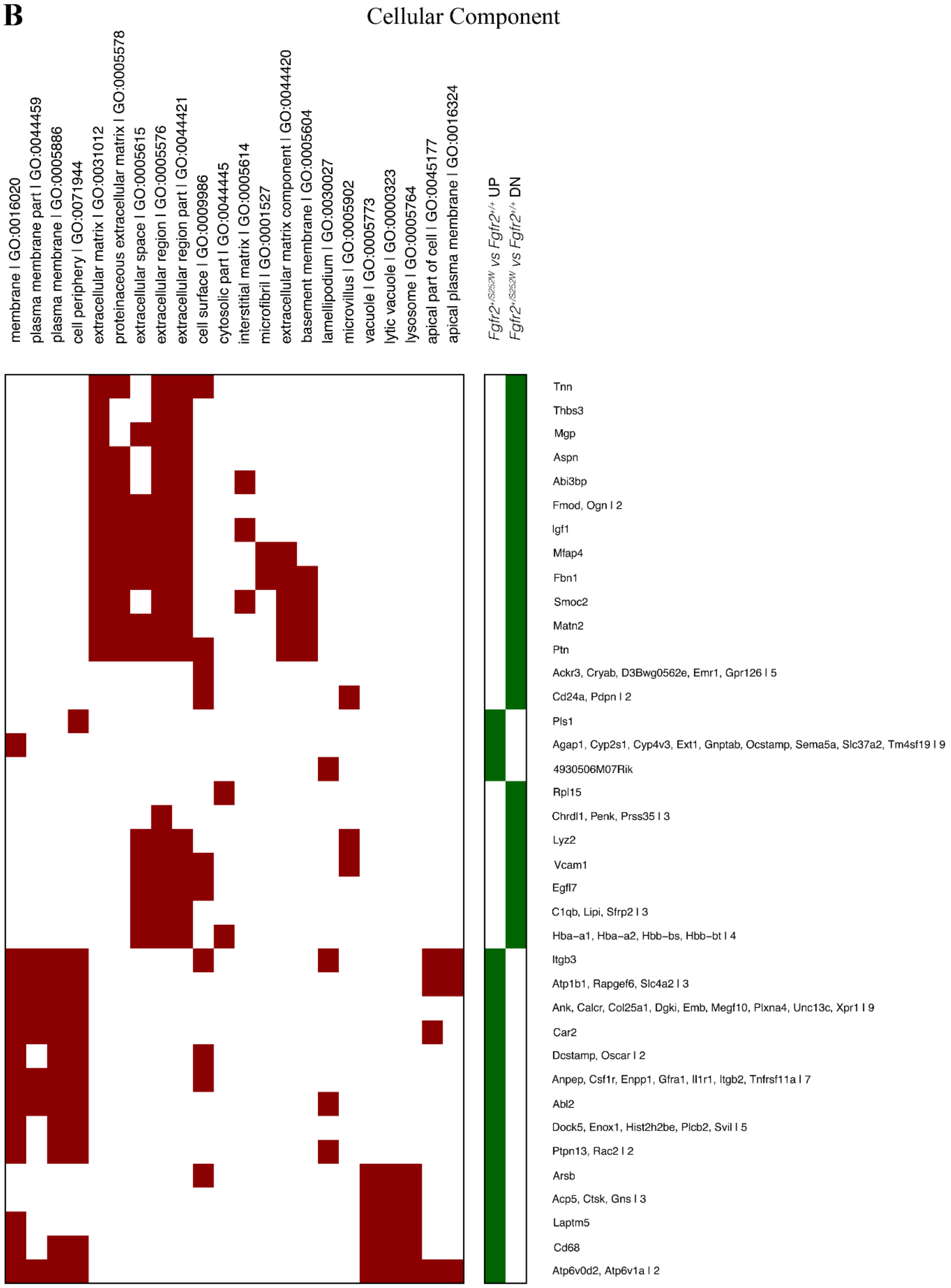

Disease Models & Mechanisms • Supplementary information

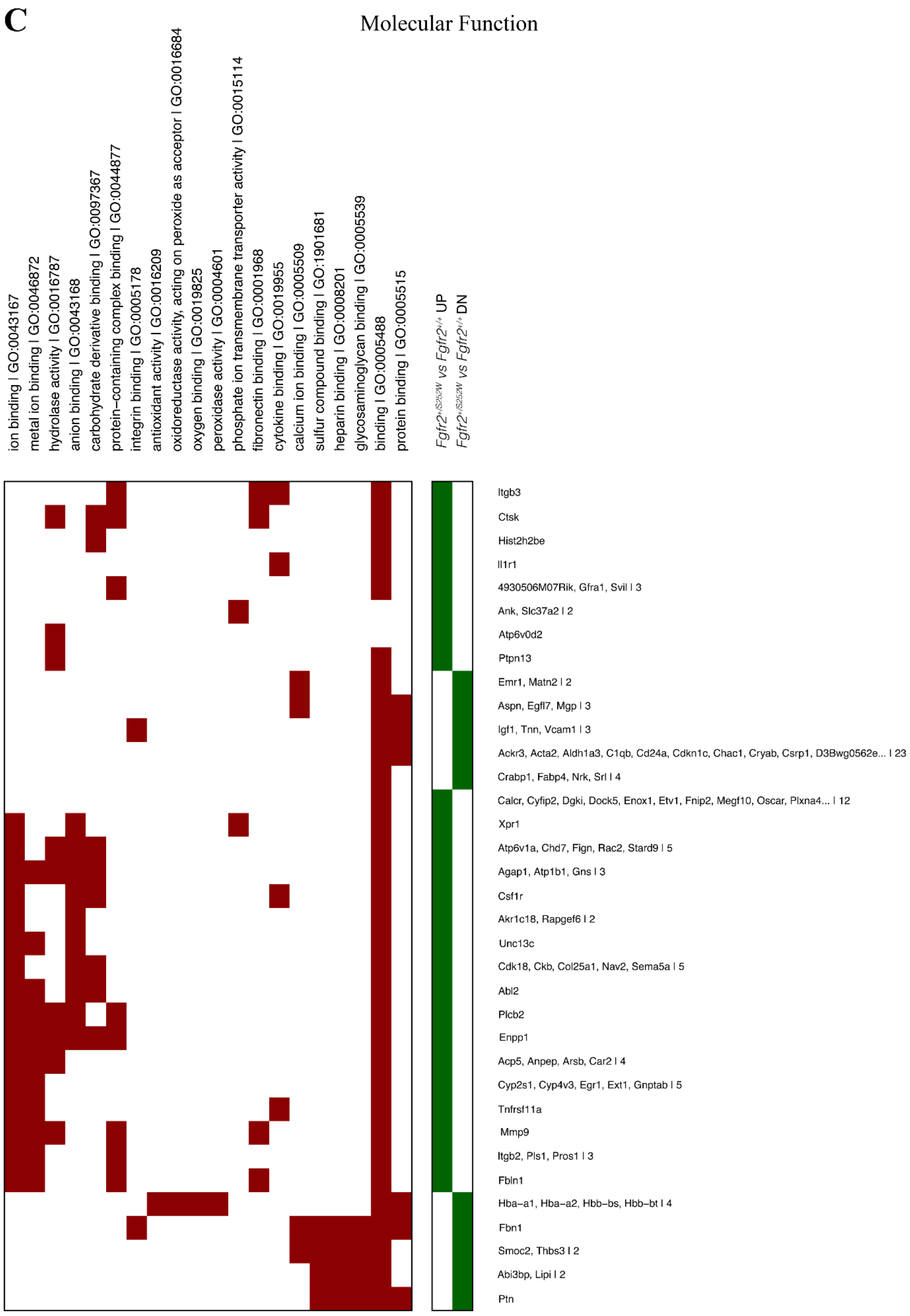

Fig. S6. Gene ontology analysis of DEGs in mandibular bone of *Fgfr2*$^{+/S252W}$ embryos at E16.5. (A) Biological process. (B) Cellular component. (C) Molecular function.

Disease Models & Mechanisms • Supplementary information

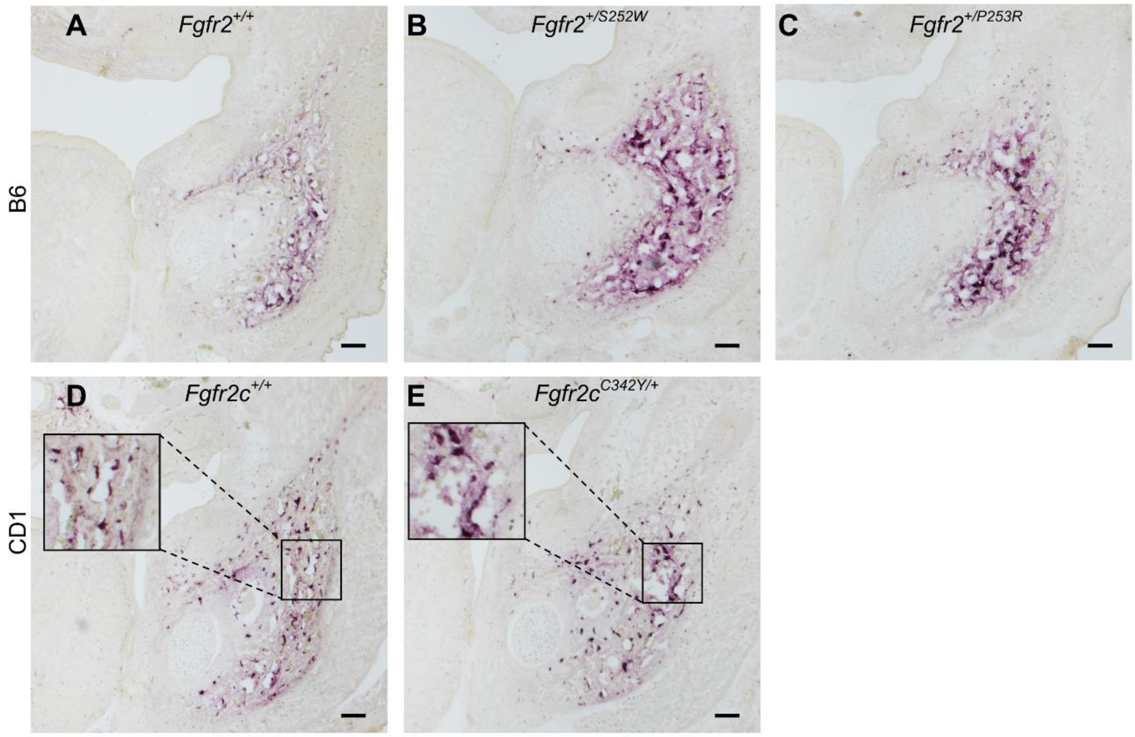

Fig. S7. Osteoclast activity in the mandibles of three FGFR2-related craniosynostosis mouse models at E16.5. (A-E) TRAP staining (purple) in mandibular bone of *Fgfr2*$^{+/+}$ (A), *Fgfr2*$^{+/S252W}$ (B) and *Fgfr2*$^{+/P253R}$ (C) embryos on a C57BL6/J (B6) background; and *Fgfr2c*$^{+/+}$ (D) and *Fgfr2c*$^{C342Y/+}$ (E) on a CD1 background. Scale bar=100 µm. The boxes in D and E indicate the area that shows increased signal in *Fgfr2c*$^{C342Y/+}$ embryos compared with unaffected littermates.

Disease Models & Mechanisms • Supplementary information

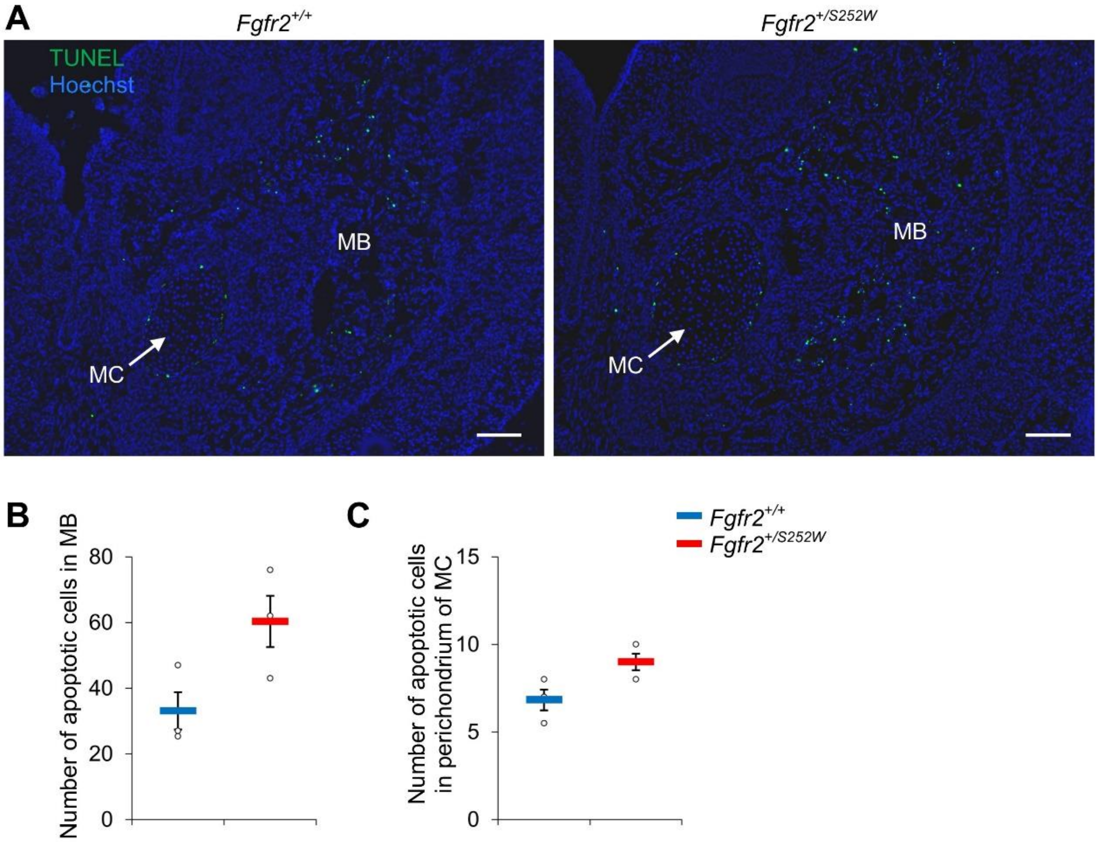

Fig. S8. Apoptosis in the mandible of *Fgfr2*$^{+/S252W}$ embryos at E16.5. (A) TUNEL assay in the mandibular area. TUNEL signal was detected in the mandibular bone (MB) and the perichondrium of Meckel's cartilage (MC) of both *Fgfr2*$^{+/+}$ and *Fgfr2*$^{+/S252W}$ embryos. Scale bar=100 µm. (B-C) Quantification shows the number of TUNEL-positive cells in the mandibular bone (B) and the perichondrium (C) of *Fgfr2*$^{+/+}$ (*n*=3) and *Fgfr2*$^{+/S252W}$ (*n*=3) embryos. The experimental data were analyzed by two-tailed Welch's t-test and expressed as the mean±s.e.m.

Disease Models & Mechanisms • Supplementary information

Table S1. Anatomical definitions of landmarks displayed in Fig. 1.

**Landmark** (Left, Right)	**Anatomical Definition**	**Region**
1, 2	Inferior-most point on incisor alveolar rim at midline of the incisor at bone-tooth junction	AB
3, 4	Junction of the rim of the alveolar process with incisor, most lateral and centered along the cranial-caudal axis (taken on bone, not tooth)	AB
5, 6	Superior-most point on incisor alveolar rim at midline of bone-tooth junction	AB
7, 8	Anterior lip of the mental foramen	AB
9, 10	Antero-lateral edge of the molar alveolar process	AB
11, 12	Intersection of molar alveolar rim and base of coronoid process (posterior molar alveolus)	R, AB
13, 14	Most dorsal point of the coronoid process	R
15, 16	Most cranio-ventral point between the coronoid and condyloid processes	R
17, 18	Most caudal point on the cranial angle of the condyloid process	R
19, 20	Most caudal point on the ventral angel of the condyloid process	R
21, 22	Most anterior point between the angle that separates the condyloid and angular processes	R
23, 24	Midpoint on the cranial caudal axis of the most posterior aspect of the angular process	R
25, 26	Cranial-most point of the angular process along the ventral surface of the mandible	R, AB
27, 28	Dorsal mandibular foramen	R
29, 30	Most ventral edge of the molar alveolar process, toward the midline	R
31, 32	Ventral mandibular foramen	AB

Landmarks are classified as located on Anterior Body (AB; n=8) or Ramus (R; n=10) to create subsets for analysis. Additional landmarks defined on the embryonic mouse skull can be found at: http://www.getahead.la.psu.edu/landmarks.

Table S2. Results (*P* values) of nonparametric null hypothesis tests for form differences (EDMA) between mice (P0) carrying a mutation for a specific craniosynostosis syndrome and their littermates without the mutation.

**Craniosynostosis Model**	**Left and Right Hemimandibles** (32 landmarks)	**Left Hemimandible** (16 landmarks)	**Right Hemimandible** (16 landmarks)
Fgfr2$^{+/S252W}$	0.001	0.001	0.001
Fgfr2$^{+/P253R}$	0.001	0.001	0.001
Fgfr2c$^{C342Y/+}$	0.001	0.001	0.001

Table S3. Differentially expressed genes in the mandibular bone of *Fgfr2*$^{+/S252W}$ embryos at E16.5 compared to their *Fgfr2*$^{+/+}$ littermates.

**Ensembl ID**	**Gene Symbol**	**log$_2$FC**	**Average Expression**	**t**	***P* Value**	**Adjusted *P* Value**
ENSMUSG00000030257	Srgap3	1.27673267	6.610930486	9.640369417	5.20142E-07	0.003057916
ENSMUSG00000037370	Enpp1	1.831773279	8.59074871	10.20061207	2.82417E-07	0.003057916
ENSMUSG00000045573	Penk	-1.928167528	4.158086959	-9.22055957	8.37469E-07	0.003188606
ENSMUSG00000034573	Ptpn13	1.133258416	6.830424831	8.998531675	1.08474E-06	0.003188606
ENSMUSG00000069917	Hba-a2	-1.622438006	4.106158794	-8.558032456	1.83915E-06	0.003902154
ENSMUSG00000022265	Ank	1.326616334	7.721340364	8.493163202	1.99123E-06	0.003902154
ENSMUSG00000073940	Hbb-bt	-1.628190337	8.121203499	-8.263608921	2.64729E-06	0.004446698
ENSMUSG00000042436	Mfap4	-1.15168023	6.836719917	-8.135654118	3.11049E-06	0.004571637
ENSMUSG00000024593	Megf10	0.927309625	6.425005115	7.528605255	6.85637E-06	0.008957467
ENSMUSG00000036905	C1qb	-2.263114164	2.013981763	-6.999425722	1.41583E-05	0.011193739
ENSMUSG00000052305	Hbb-bs	-1.57470114	9.839937637	-7.157779	1.13549E-05	0.011193739
ENSMUSG00000035783	Acta2	-1.163348891	5.560032653	-6.993321688	1.42802E-05	0.011193739
ENSMUSG00000006369	Fbln1	0.877748065	5.903300431	7.109264313	1.21449E-05	0.011193739
ENSMUSG00000054594	Oscar	1.217936999	5.141501095	7.270388572	9.72461E-06	0.011193739
ENSMUSG00000040703	Cyp2s1	1.331780479	3.815520063	6.998767749	1.41714E-05	0.011193739
ENSMUSG00000031351	Zfp185	-2.478906784	3.159340528	-6.742466291	2.03933E-05	0.011887187
ENSMUSG00000033491	Prss35	-1.351275459	5.889560643	-6.721057207	2.1031E-05	0.011887187
ENSMUSG00000040569	Slc26a7	-1.106708548	6.18519432	-6.763093111	1.97983E-05	0.011887187
ENSMUSG00000037664	Cdkn1c	-0.880989857	8.705576379	-6.714493501	2.12307E-05	0.011887187
ENSMUSG00000024621	Csf1r	0.932343075	7.271710056	6.790660552	1.90318E-05	0.011887187
ENSMUSG00000020340	Cyfip2	1.222456601	6.830403391	6.838067414	1.77866E-05	0.011887187
ENSMUSG00000028047	Thbs3	-1.146915292	4.350320931	-6.66151792	2.29185E-05	0.012248919
ENSMUSG00000052512	Nav2	0.954836312	6.560031841	6.618351607	2.43993E-05	0.012473328
ENSMUSG00000014846	Tppp3	-2.657022312	2.498697296	-6.399689044	3.36333E-05	0.015732053
ENSMUSG00000055013	Agap1	0.824170057	6.949305293	6.376950077	3.47877E-05	0.015732053
ENSMUSG00000023964	Calcr	1.39086817	4.385746777	6.386112042	3.43176E-05	0.015732053
ENSMUSG00000044667	Plppr4	-1.64880105	3.281452634	-6.337299974	3.69024E-05	0.016070294
ENSMUSG00000021388	Aspn	-1.565468808	7.851433763	-6.235000826	4.30138E-05	0.017532802
ENSMUSG00000026321	Tnfrsf11a	0.878575936	6.328271017	6.231469435	4.3243E-05	0.017532802

ENSMUSG00000069516	Lyz2	-1.48397449	5.657315666	-5.984140315	6.30167E-05	0.021283798
ENSMUSG00000030218	Mgp	-1.221549215	4.443285577	-6.029622637	5.87632E-05	0.021283798
ENSMUSG00000041559	Fmod	-0.935657006	6.937668196	-6.047249988	5.71975E-05	0.021283798
ENSMUSG00000026469	Xpr1	0.756418685	8.588488909	5.965430507	6.48599E-05	0.021283798
ENSMUSG00000035311	Gnptab	0.877553017	7.078081271	6.048247317	5.71102E-05	0.021283798
ENSMUSG00000022303	Dcstamp	1.019517844	5.581854423	5.945315992	6.69054E-05	0.021283798
ENSMUSG00000040061	Plcb2	1.240168118	3.840512082	5.944636192	6.69757E-05	0.021283798
ENSMUSG00000021214	Akr1c18	1.299377329	4.04982874	5.9744297	6.39663E-05	0.021283798
ENSMUSG00000020053	Igf1	-1.273626277	6.98869413	-5.913249668	7.03087E-05	0.021754992
ENSMUSG00000028238	Atp6v0d2	0.857306076	8.426331011	5.891337145	7.27394E-05	0.021929993
ENSMUSG00000031283	Chrdl1	-0.834377044	5.949540909	-5.813408207	8.21296E-05	0.021947271
ENSMUSG00000001270	Ckb	0.899333325	8.11410426	5.841953781	7.85492E-05	0.021947271
ENSMUSG00000050390	C77080	1.100606755	4.008047025	5.824856679	8.06733E-05	0.021947271
ENSMUSG00000058897	Col25a1	1.145484143	6.457814805	5.817728948	8.15768E-05	0.021947271
ENSMUSG00000025089	Gfra1	1.467929152	4.267875815	5.853457969	7.71533E-05	0.021947271
ENSMUSG00000012405	Rpl15	-0.906856775	6.677200075	-5.773069762	8.74862E-05	0.022362233
ENSMUSG00000021728	Emb	0.914199758	5.859278388	5.781838677	8.62911E-05	0.022362233
ENSMUSG00000032948	Lipi	-0.936729854	5.444723773	-5.663403914	0.000104002	0.025438301
ENSMUSG00000022324	Matn2	-0.694566276	5.740143219	-5.640097671	0.000107918	0.025438301
ENSMUSG00000052459	Atp6v1a	0.718305056	8.851933228	5.638604413	0.000108174	0.025438301
ENSMUSG00000044447	Dock5	1.048477353	6.571337781	5.639942804	0.000107945	0.025438301
ENSMUSG00000017737	Mmp9	1.19764316	9.755281083	5.569229207	0.000120814	0.027853587
ENSMUSG00000035296	Sgcg	-4.411710792	-0.922779598	-5.517657881	0.000131215	0.028010594
ENSMUSG00000032291	Crabp1	-2.155264427	3.115239671	-5.497116825	0.000135618	0.028010594
ENSMUSG00000052854	Nrk	-1.487662892	6.623819756	-5.547251564	0.000125136	0.028010594
ENSMUSG00000001348	Acp5	0.793398575	8.627342467	5.516153703	0.000131533	0.028010594
ENSMUSG00000021306	Gpr137b	0.861604003	6.046324047	5.519626396	0.000130802	0.028010594
ENSMUSG00000032122	Slc37a2	1.191073369	7.077239901	5.496333193	0.000135789	0.028010594
ENSMUSG00000069919	Hba-a1	-1.337990689	7.815219545	-5.422264917	0.000153024	0.030909916
ENSMUSG00000020689	Itgb3	1.110567359	7.880369008	5.41393388	0.000155102	0.030909916
ENSMUSG00000027562	Car2	0.745533042	6.189698692	5.394819185	0.000159983	0.031351269
ENSMUSG00000022519	Srl	-0.874711972	4.426953711	-5.369870117	0.000166598	0.03211249
ENSMUSG00000040037	Negr1	-2.06759258	3.207329165	-5.347098779	0.000172888	0.032266944
ENSMUSG00000041362	Shtn1	0.856606818	5.623009126	5.348913307	0.000172378	0.032266944
ENSMUSG00000029765	Plxna4	1.049654389	5.846000814	5.319457787	0.000180861	0.033227474
ENSMUSG00000035258	Abi3bp	-1.564181951	4.695670152	-5.29502116	0.000188231	0.033533688
ENSMUSG00000027962	Vcam1	-1.033135854	4.531254283	-5.295700595	0.000188022	0.033533688
ENSMUSG00000021390	Ogn	-1.35118064	7.552490868	-5.285796718	0.000191095	0.033535785
ENSMUSG00000047139	Cd24a	-0.988039433	5.207433327	-5.274863718	0.000194549	0.033639831
ENSMUSG00000026072	Il1r1	0.782735636	4.957639421	5.248885822	0.000203022	0.034101898
ENSMUSG00000028581	Laptm5	0.879244011	6.642567459	5.25154349	0.000202138	0.034101898
ENSMUSG00000032060	Cryab	-1.110150001	3.610742127	-5.220757626	0.000212635	0.035213634
ENSMUSG00000024236	Svil	0.624813843	7.133248611	5.188180982	0.000224371	0.036640971
ENSMUSG00000026576	Atp1b1	1.17396213	4.596809676	5.172977602	0.000230079	0.037058409
ENSMUSG00000042082	Arsb	0.97021764	7.506223253	5.160310942	0.000234951	0.037331753
ENSMUSG00000004151	Etv1	0.730282583	4.988104973	5.132028881	0.000246224	0.038601426
ENSMUSG00000002014	Ssr4	-0.810564086	5.903376453	-5.097609777	0.000260716	0.039301265
ENSMUSG00000034707	Gns	0.727994379	7.459110088	5.104733751	0.000257645	0.039301265
ENSMUSG00000027670	Ocstamp	1.019536701	5.872442547	5.107174041	0.000256601	0.039301265
ENSMUSG00000061731	Ext1	0.663550112	7.436421895	5.069385813	0.000273267	0.04067181
ENSMUSG00000035357	Pdzrn3	-0.775776383	5.557112496	-5.047366705	0.000283499	0.041667193
ENSMUSG00000000290	Itgb2	0.939153536	5.118505724	5.024342179	0.000294628	0.042768391
ENSMUSG00000033220	Rac2	0.7747666	6.466527167	5.001754487	0.000305993	0.043876437
ENSMUSG00000023886	Smoc2	-1.076440655	5.017467359	-4.98683623	0.000313751	0.043917621
ENSMUSG00000039116	Gpr126	-0.981742058	4.952859094	-4.988178407	0.000313044	0.043917621
ENSMUSG00000015134	Aldh1a3	-1.172030326	5.661165159	-4.96080253	0.000327786	0.044910263
ENSMUSG00000026921	Egfl7	-0.844921515	4.284607323	-4.957154752	0.000329805	0.044910263
ENSMUSG00000018567	Gabarap	-0.694331619	7.756610472	-4.952676418	0.000332301	0.044910263
ENSMUSG00000037533	Rapgef6	0.614649699	7.303891696	4.92217191	0.000349838	0.046743173

Disease Models & Mechanisms • Supplementary information

ENSMUSG00000026596	Abl2	0.608326336	6.50724323	4.910707644	0.000356678	0.047121542
ENSMUSG00000044468	Fam46c	-0.684580972	6.669624759	-4.897416701	0.000364783	0.047656834
ENSMUSG00000004730	Emr1	-2.239807676	1.348603199	-4.832587645	0.000407175	0.049783334
ENSMUSG00000027313	Chac1	-2.213689517	4.887600415	-4.764393172	0.000457375	0.049783334
ENSMUSG00000027996	Sfrp2	-1.171324394	4.710267004	-4.788885324	0.000438639	0.049783334
ENSMUSG00000062515	Fabp4	-1.05622216	3.509023446	-4.704234021	0.000507036	0.049783334
ENSMUSG00000028583	Pdpn	-1.045235352	3.565172539	-4.802701535	0.000428426	0.049783334
ENSMUSG00000044337	Ackr3	-1.019213136	4.030412869	-4.708330855	0.000503482	0.049783334
ENSMUSG00000029838	Ptn	-0.936047162	8.138301746	-4.752584264	0.000466706	0.049783334
ENSMUSG00000027204	Fbn1	-0.801254068	6.408244189	-4.808538301	0.000424186	0.049783334
ENSMUSG00000026421	Csrp1	-0.783880408	6.759861112	-4.787747037	0.000439492	0.049783334
ENSMUSG00000026725	Tnn	-0.777064263	7.437115497	-4.847170068	0.000397209	0.049783334
ENSMUSG00000071856	Mcc	-0.73828315	5.345518229	-4.714396914	0.000498266	0.049783334
ENSMUSG00000024534	Sncaip	-0.723674183	4.868083805	-4.694867838	0.000515261	0.049783334
ENSMUSG00000038871	Bpgm	-0.682666203	5.249711873	-4.7094474	0.000502517	0.049783334
ENSMUSG00000022912	Pros1	0.663131966	6.459136494	4.749861565	0.000468885	0.049783334
ENSMUSG00000033705	Stard9	0.706268577	6.374308981	4.698304549	0.000512227	0.049783334
ENSMUSG00000061175	Fnip2	0.710174275	5.074804605	4.84910423	0.000395907	0.049783334
ENSMUSG00000028111	Ctsk	0.713395398	9.9623244	4.816083029	0.000418771	0.049783334
ENSMUSG00000075324	Fign	0.737656804	5.609855492	4.780463665	0.000444989	0.049783334
ENSMUSG00000028962	Slc4a2	0.773555819	6.05593102	4.795937482	0.000433394	0.049783334
ENSMUSG00000041235	Chd7	0.793445297	5.723571848	4.736175534	0.000480003	0.049783334
ENSMUSG00000039062	Anpep	0.813927796	8.056912398	4.76404628	0.000457646	0.049783334
ENSMUSG00000068854	Hist2h2be	0.830691553	4.786180283	4.735427416	0.000480619	0.049783334
ENSMUSG00000038665	Dgki	0.831832322	4.196277449	4.707501683	0.000504199	0.049783334
ENSMUSG00000079057	Cyp4v3	0.83836864	4.764351948	4.838702637	0.000402965	0.049783334
ENSMUSG00000022231	Sema5a	0.839094343	9.365098271	4.837629137	0.0004037	0.049783334
ENSMUSG00000026437	Cdk18	0.88555389	3.867251827	4.693416906	0.000516548	0.049783334
ENSMUSG00000022012	Enox1	0.892698352	3.686678832	4.709026013	0.000502881	0.049783334
ENSMUSG00000018774	Cd68	0.959283668	7.245963357	4.703581561	0.000507605	0.049783334
ENSMUSG00000079625	Tm4sf19	1.030522587	3.924772294	4.762574467	0.000458799	0.049783334
ENSMUSG00000038418	Egr1	1.120357726	6.447009919	4.772930692	0.000450751	0.049783334
ENSMUSG00000049493	Pls1	1.373575186	2.360582503	4.733077184	0.000482558	0.049783334
ENSMUSG00000062151	Unc13c	1.540649612	3.574785275	4.723859106	0.000490244	0.049783334

Table S4. Primers used to generate riboprobe templates for RNA *in situ* hybridization.

Gene	Primers	References
Ank	5'-GAGTAATACGACTCACTATAGGGTGGGATGTGCCTCAATCTCA-3'<br>5'-GAGATTAACCCTCACTAAAGGGACACAGAGTTCTGCAAAGGCAA-3'	Uzuki et al., 2014
Csf1r	5'-GAGTAATACGACTCACTATAGGGAGGAGGTGTCTGTGGGTGAC-3'<br>5'-GAGATTAACCCTCACTAAAGGGATGGTACTTCGGCTTCTGCTT-3'	Designed using Primer3
Enpp1	5'-GAGTAATACGACTCACTATAGGGGCTGTCTGAGACTCCCTTGG-3'<br>5'-GAGATTAACCCTCACTAAAGGGAGTCCCCAGACCACGTACACT-3'	Designed using Primer3
Itgb3	5'-GAGTAATACGACTCACTATAGGGGAAAATGTCGTCAGCCTTTACC-3'<br>5'-GAGATTAACCCTCACTAAAGGGAGCAGGAGAAGTCATCGCACTC-3'	Diez-Roux et al., 2011

Disease Models & Mechanisms • Supplementary information
